# Reduced meat and dairy consumption improves health, environmental and most nutritional outcomes without increasing diet costs among Scottish adults

**DOI:** 10.1038/s43016-026-01384-3

**Published:** 2026-07-03

**Authors:** Joe Kennedy, Michael Clark, Cristina Stewart, Ricki Runions, Alexander Vonderschmidt, Sarah M. Frank, Peter Scarborough, Fiona Comrie, Alana McDonald, Geraldine McNeill, Peter Alexander, Lindsay M. Jaacks

**Affiliations:** 1 Division of Global Agriculture and Food Systems, The University of Edinburgh, Midlothian, UK; 2 Smith School of Enterprise and the Environment, University of Oxford, Oxford, UK; 3 Oxford Martin School, University of Oxford, Oxford, UK; 4 Department of Biology, University of Oxford, Oxford, UK; 5 School of Health and Wellbeing, University of Glasgow, Glasgow, UK; 6 Food is Medicine Institute, Friedman School of Nutrition Science and Policy, Tufts University, Boston, MA USA; 7 Nuffield Department of Primary Care Health Sciences, NIHR Oxford Health Biomedical Research Centre, University of Oxford, Oxford, UK; 8 Food Standards Scotland, Aberdeen, UK; 9 School of Geosciences, University of Edinburgh, Edinburgh, UK

**Keywords:** Environmental sciences, Cardiovascular diseases, Diabetes, Risk factors

## Abstract

Shifting diets away from high levels of meat and dairy is increasingly considered an important part of climate mitigation, yet the best pathways for achieving these reductions without compromising nutrition, health or affordability remain unclear. Here, in a representative sample of Scottish adults, we evaluate 33 pathways to meeting the UK Climate Change Committee’s recommendations to reduce meat and dairy consumption by 20% by 2030, increasing to a 35% reduction in meat by 2050. The pathways incorporate existing dietary guidance, and modelled outcomes include intakes of 54 nutrients, obesity, type 2 diabetes, cardiovascular disease, all-cause mortality, diet costs, greenhouse gas emissions, freshwater use, land use and eutrophication. Nearly all pathways were estimated to benefit most nutritional, health and environmental outcomes without increasing diet costs. Benefits were greater when reductions targeted high consumers of red meat and when meat and dairy were replaced gram for gram with foods such as vegetables, beans, eggs and plant-based dairy alternatives.

## Abstract (web summary)

Using microsimulation of Scottish Health Survey adults, this study compares 33 pathways for achieving the UK Climate Change Committee meat and dairy reduction targets. Most pathways improved nutrient intake, health and environmental outcomes without increasing diet costs, with greater benefits from reducing red and processed meat among high consumers and using gram-for-gram substitutions with foods including vegetables, beans, eggs and plant-based alternatives.

## Main

Reducing the consumption of animal source foods has been recommended in high-consuming countries to help meet environmental targets^1^. In the UK, the Climate Change Committee (CCC) has recommended a reduction in all meat and dairy consumption by 20% by 2030, increasing to a reduction of 35% in meat consumption by 2050^2^. However, dietary guidance in the UK, known as the Eatwell Guide, focuses specifically on eating less red and red processed meat^3^, given evidence of an association between regular consumption of high levels of red and processed meat with increased risk of developing several chronic diseases^4,5^. The Scottish Dietary Goals set a recommended maximum intake of red and red processed meat at 70 g per day^6^. There is a gap in understanding the most effective pathway to meeting the UK CCC dietary recommendations, taking into account existing dietary guidance, impacts on nutrient intake, chronic disease risk, greenhouse gas (GHG) emissions as well as other environmental outcomes such as water and land use, and eutrophication. The transition to net zero must be affordable to avoid perpetuating or exacerbating health inequalities, and so pathways should also account for the cost of dietary transitions, particularly for low-income households.

In Scotland, where the CCC dietary recommendations have been ‘partially accepted’^7^, no study has explored the impact of meat and dairy reductions on nutrient intake, chronic disease risk, the environment and cost of diets overall or in specific population subgroups—especially among those living in the most deprived areas. UK-wide studies have generally estimated the effect in the whole population, modelling the impact of a reduction in meat replaced with fruit, vegetables, legumes and/or cereals on nutrient intake, mortality and/or GHG emissions^8–12^. Comparatively few studies have evaluated concurrent reductions in dairy, the impact on the cost of diets, variations in impacts by population subgroup or replacement with plant-based meat and dairy alternatives^9,13,14^.

In the present study, we employed a microsimulation, which enables estimation by population subgroup, to quantify how different pathways to achieving the CCC recommendations affected dietary intake of 54 nutrients; the prevalence of obesity and the incidence of type 2 diabetes, cardiovascular disease and all-cause mortality; cost of diets; and GHG emissions, freshwater use (herein water use), land use and eutrophication. Dietary intake, including nutrient intake, and health data were from the latest round of the Scottish Health Survey (SHeS 2021), which is designed to be representative and included adults 16 years and older. Data on environmental impact and diet cost were taken from foodDB, a product-specific dataset containing the estimated environmental impact and price of approximately 70,000 items available in UK supermarkets^15,16^, which we matched to dietary intake data reported in SHeS. We also provide estimates for the population-level yearly dietary emissions adjusted for underreported caloric intake in SHeS 2021.

We evaluated 33 meat and dairy reduction pathways (Table 1). Pathways focused on reductions in all meat (beef, lamb, pork and poultry; herein ‘meat’) as specified in the CCC dietary recommendations, or reductions in red and red processed meat (beef, lamb and pork; herein ‘red meat’) among high consumers as specified in the Scottish Dietary Goals, together with reductions in all dairy. ‘High consumers’ were those consuming above the maximum threshold for red meat—either 70 g per day, 60 g per day or 31 g per day, depending on the pathway. To help interpret the combined effects of meat and dairy reductions, we ran two additional pathways: a 20% reduction in dairy with no reduction in meat consumption, both with and without a plant-based dairy alternative substitution. We did not consider maximum intake thresholds for dairy as there is no such guideline in the Scottish Dietary Goals. For each meat and dairy reduction pathway, we considered gram-for-gram replacement of meat with beans and pulses, vegetables, eggs, non-smoked oily fish, plant-based meat alternatives or (for red meat reduction pathways only) poultry; and a gram-for-gram replacement of dairy with plant-based milks, yoghurts and solid fats and no replacement for cheese. We chose gram-for-gram replacements because a calorie-for-calorie replacement would be more difficult for individuals to adopt given limited consumer knowledge and understanding of calories^17^. While we provide the results for health outcomes assuming an isocaloric substitution in Supplementary Information, gram-for-gram replacement of meat with other foods may result in a modest decrease in caloric intake and associated greater health benefits compared with the isocaloric results. We did not consider plant-based cheeses given limited availability in the Scottish food retail environment^18^.

**Table 1:** Summary of meat and dairy reduction pathways and outcomes assessed

	Pathway name	Reduction in meat	Reduction in dairy	Replacement of meat	Replacement of dairy	Outcomes assessed			
						Nutrient intake	Environment^a^	Cost of diets	Health^b^
1	CCC 2030, no replacement	20% reduction in all meat	20% reduction in all dairy	None	None	✓	✓	✓	✓
2	CCC 2030, beans and PBDA			Beans/pulses	PBDA^c^	✓	✓	✓	
3	CCC 2030, vegetables and PBDA			Vegetables		✓	✓	✓	
4	CCC 2030, eggs and PBDA			Eggs		✓	✓	✓	
5	CCC 2030, fish and PBDA			Non-smoked oily fish		✓	✓	✓	
6	CCC 2030, PBMA and PBDA			PBMA		✓	✓	✓	
7	CCC 2050, no replacement	35% reduction in all meat	20% reduction in all dairy	None	None	✓	✓	✓	✓
8	CCC 2050, beans and PBDA			Beans/pulses	PBDA^c^	✓	✓	✓	
9	CCC 2050, vegetables and PBDA			Vegetables		✓	✓	✓	
10	CCC 2050, eggs and PBDA			Eggs		✓	✓	✓	
11	CCC 2050, fish and PBDA			Non-smoked oily fish		✓	✓	✓	
12	CCC 2050, PBMA and PBDA			PBMA		✓	✓	✓	
13	SDG, no replacement	Red meat reduced to max 70 g per day^d^	20% reduction in all dairy	None	None	✓	✓	✓	✓
14	SDG, beans and PBDA			Beans and pulses	PBDA^c^	✓	✓	✓	
15	SDG, vegetables and PBDA			Vegetables		✓	✓	✓	
16	SDG, eggs and PBDA			Eggs		✓	✓	✓	
17	SDG, fish and PBDA			Non-smoked oily fish		✓	✓	✓	
18	SDG, PBMA and PBDA			PBMA		✓	✓	✓	
19	SDG, poultry and PBDA			Poultry		✓	✓	✓	
20	Red meat 60 g per day max, no replacement	Red meat reduced to max 60 g per day^e^	20% reduction in all dairy	None	None	✓	✓	✓	✓
21	Red meat 60 g per day max, beans and PBDA			Beans and pulses	PBDA^c^	✓	✓	✓	
22	Red meat 60 g per day max, vegetables and PBDA			Vegetables		✓	✓	✓	
23	Red meat 60 g per day max, eggs and PBDA			Eggs		✓	✓	✓	
24	Red meat 60 g per day max, fish and PBDA			Non-smoked oily fish		✓	✓	✓	
25	Red meat 60 g per day max, PBMA and PBDA			PBMA		✓	✓	✓	
26	Red meat 60 g per day max, poultry and PBDA			Poultry		✓	✓	✓	
27	Red meat 31 g per day max, no replacement	Red meat reduced to max 31 g per day^f^	20% reduction in all dairy	None	None	✓	✓	✓	✓
28	Red meat 31 g per day max, beans and PBDA			Beans and pulses	PBDA^c^	✓	✓	✓	
29	Red meat 31 g per day max, vegetables and PBDA			Vegetables		✓	✓	✓	
30	Red meat 31 g per day max, eggs and PBDA			Eggs		✓	✓	✓	
31	Red meat 31 g per day max, fish and PBDA			Non-smoked oily fish		✓	✓	✓	
32	Red meat 31 g per day max, PBMA and PBDA			PBMA		✓	✓	✓	
33	Red meat 31 g per day max, poultry and PBDA			Poultry		✓	✓	✓	
34	20% dairy reduction, no replacement	None	20% reduction in all dairy	None	None	✓	✓	✓	✓
35	20% dairy reduction, PBDA	None	20% reduction in all dairy	None	PBDA^c^	✓	✓	✓	

All meat includes beef, lamb, pork, processed red meat, other red meat, burgers, sausages, offal, poultry, processed poultry and game birds. Red meat includes beef, lamb, pork, processed red meat, other red meat, burgers, sausages and offal. Replacements of meat and dairy were gram for gram.

PBDA, plant-based dairy alternatives; PBMA, plant-based meat alternatives; SDG, Scottish Dietary Goal.

^a^Environmental outcomes included GHG emissions, water and land use, and eutrophication.

^b^Health outcomes included prevalence of obesity, the incidence of type 2 diabetes, cardiovascular disease and all-cause mortality.

^c^Plant-based dairy alternatives included plant-based milks, yoghurts and solid fats but not cheese given limited availability in the Scottish food retail environment.

^d^Scottish Dietary Goal for red meat.

^e^60 g per day is the amount of reduction in red meat that would achieve an overall reduction in all meat of 20%, the CCC target for 2030.

^f^31 g per day is the amount of reduction in red meat that would achieve an overall reduction in all meat of 35%, the CCC target for 2050.

Given the large number of pathways (35 total) and outcomes (63 total) evaluated, overall and by population subgroup (sex, 10-year age groups (16–24 years, 25–34 years, 35–44 years and so on) and Scottish Index of Multiple Deprivation (SIMD) quintiles), we chose to highlight specific pathways and outcomes in the text. Results for all outcomes for all reduction pathways by population subgroups are provided in Supplementary Data. In the text, we highlight pathway 27—a reduction in red meat to a maximum of 31 g per day together with a 20% reduction in all dairy, and no replacement—as the ‘most effective pathway’ because this pathway resulted in the greatest benefits for health and the environment together with dietary cost savings. Impacts on intakes of calcium, iodine, zinc and selenium are highlighted because meat and dairy were previously identified as important sources of these nutrients in Scottish diets^19^. We further highlight impacts on cardiovascular disease by population subgroup because cardiovascular disease is the leading cause of death in Scotland^20^.

## Results

### Baseline diets

**Fig. 1:**
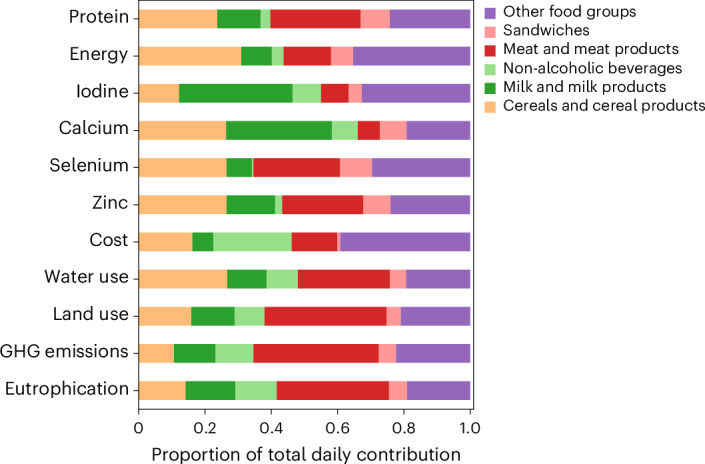
Food category relative contributions at baseline. Relative contribution of each food category to intake of select nutrients, food costs and environmental indicators in a representative sample of adults 16+ years living in Scotland (2021) (*n* = 3,447).

In 2021, total GHG emissions associated with food consumed by adults living in Scotland were estimated to be 10.4 Mt CO_2_ equivalents (CO_2_e) (95% uncertainty interval (UI), 10.3 to 10.6 MtCO_2_e) after adjusting for underreported daily caloric intake (Supplementary Information p. 17). Mean unadjusted (95% UI) per capita daily GHG emissions were 4.34 kgCO_2_e (4.24 to 4.44 kgCO_2_e), and the cost of diets was £8.88 (£8.65 to £9.10). Meat and meat products were the largest contributor to each environmental outcome, accounted for 14% of the cost of diets and were responsible for approximately a quarter of protein, zinc and selenium intake. Milk and milk products accounted for 6% of diet cost, 10–15% of each environmental outcome, and a third of iodine and calcium intake (Fig. 1 and Supplementary Data).

### Health and nutritional outcomes

**Fig. 2:**
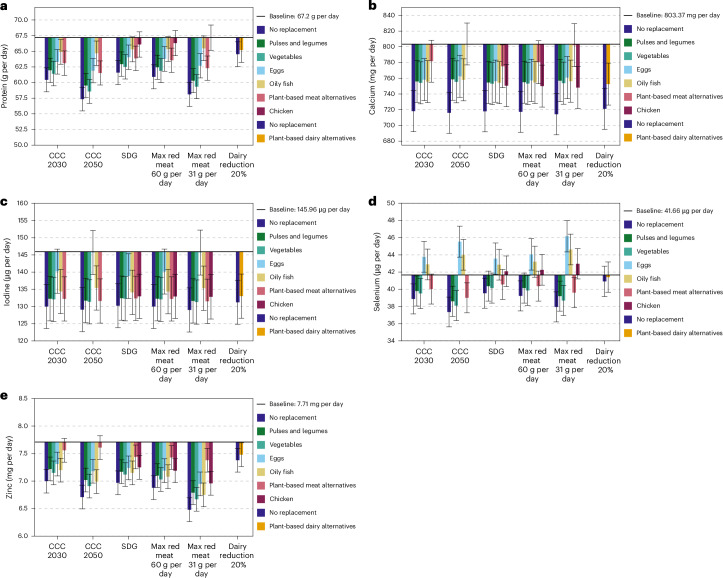
Impact on nutrient intake in each pathway. **a**–**e**, Impact on per capita intake of protein (g per day) (**a**), calcium (mg per day) (**b**), iodine (µg per day) (**c**), selenium (µg per day) (**d**) and zinc (mg per day) (**e**) in each meat and dairy reduction pathway in a representative sample of adults 16+ years living in Scotland (*n* = 3,447). Each bar represents the difference between the mean per capita intake at baseline and each pathway. Error bars represent 95% UIs, calculated as 1.96 multiplied by the standard error of the difference in intake at baseline and each pathway.

In the most effective pathway (pathway 27), the average drop in daily protein intake was 9.1 g, or 13.5%, compared with an average per capita daily intake at baseline of 67.2 g (Table 2 and Fig. 2a). The decrease in calcium intake in the pathways with no replacement was largely driven by the decrease in dairy, with 92% of the decrease in calcium intake in the most effective pathway resulting from the dairy reduction, corresponding to an ~10% decrease in per capita calcium intake. The age group 16–24 years started with the lowest baseline per capita intake of all other age groups at an average of 749 mg per day, which decreased to 665 mg per day in the most effective pathway. However, this decrease in calcium intake was compensated in all age groups when red and red processed meat was replaced with plant-based meat alternatives (Fig. 2b and Supplementary Data). The decrease in iodine intake was also driven by the decrease in dairy, with 87% of the decrease in iodine intake in the most effective pathway arising from dairy reduction corresponding to an ~11% decrease in per capita iodine intake. Given the lack of iodine in the oat milk represented in the UK nutrient databank, which made up ~50% of the plant-based replacement for dairy milk (Supplementary Information p. 8), replacement of dairy with plant-based milks, yogurts and solid fats did not substantially increase iodine intake (Fig. 2c).

**Table 2:** Mean (95% UI) impact of reducing meat and dairy from baseline (2021) levels with no replacement on nutrient intake, environmental outcomes, cost of diets and chronic disease in a representative sample of adults 16+ years living in Scotland

Pathway^a^	CCC 2030	CCC 2050	Scottish Dietary Goal (red meat 70 g per day max)	Red meat 60 g per day max	Red meat 31 g per day max	Dairy reduction, 20%
**Change in per capita nutrient intake**						
Protein, g per day	−6.77 (−8.69 to −4.85)	−9.85 (−11.73 to −7.97)	−5.61 (−7.53 to −3.69)	−6.29 (−8.21 to −4.37)	−9.09 (−11.01 to −7.18)	−2.67 (−4.65 to −0.69)
Zinc, mg per day	−0.71 (−0.93 to −0.49)	−1.00 (−1.22 to −0.78)	−0.74 (−0.96 to −0.52)	−0.83 (−1.04 to −0.62)	−1.23, (−1.45 to −1.01)	−0.33 (−0.55 to −0.11)
Calcium, mg per day	−85.17 (−111.32 to −59.02)	−87.36 (−113.53 to −61.19)	−85.36 (−111.45 to −59.27)	−86.08 (−112.19 to −59.97)	−89.09 (−115.24 to −62.94)	−82.24 (−108.37 to −56.11)
Iodine, µg per day	−15.96 (−22.35 to −9.57)	−16.87 (−23.28 to −10.46)	−15.72 (−22.11 to −9.33)	−15.96 (−22.35 to −9.57)	−16.96 (−23.37 to −10.55)	−14.74 (−21.13 to −8.35)
Selenium, µg per day	−2.78 (−4.52 to −1.04)	−4.30 (−6.02 to −2.58)	−2.12 (−3.86 to −0.38)	−2.43 (−4.17 to −0.69)	−3.71 (−5.45 to −1.97)	−0.74 (−2.5 to 1.02)
**Change in per capita consumption impacts**						
GHG emissions, kgCO_2_e per day	−0.47 (−0.59 to −0.35)	−0.71 (−0.83 to −0.59)	−0.49 (−0.61 to −0.37)	−0.57 (−0.69 to −0.45)	−0.91, (−1.03 to −0.79)	−0.15 (−0.27 to −0.03)
Land use, m^2^ per day	−0.61 (−0.79 to −0.43)	−0.94 (−1.12 to −0.76)	−0.66 (−0.84 to −0.48)	−0.77 (−0.95 to −0.59)	−1.25 (−1.41 to −1.09)	−0.18 (−0.38 to 0.02)
Eutrophication, gPO_4_e per day	−1.85 (−2.3 to −1.4)	−2.68 (−3.11 to −2.25)	−1.94 (−2.37 to −1.51)	−2.23 (−2.66 to −1.8)	−3.47 (−3.9 to −3.04)	−0.72 (−1.19 to −0.25)
Water use, litres per day	−39.66 (−57.54 to −21.78)	−58.22 (−76.0 to −40.44)	−42.18 (−59.94 to −24.42)	−48.52 (−66.26 to −30.78)	−75.24 (−93.0 to −57.48)	−14.78 (−32.87 to 3.31)
**Change in per capita price**						
£ per day	−0.29 (−0.60 to 0.03)	−0.41 (−0.72 to −0.10)	−0.25 (−0.56 to 0.07)	−0.28 (−0.59 to 0.04)	−0.41 (−0.72 to −0.09)	−0.12 (−0.43 to 0.20)
**Health outcomes over 10 years**						
Prevented type 2 diabetes cases	36,491 (30,906 to 42,098)	49,179 (41,122 to 56,906)	38,970 (32,571 to 45,172)	43,162 (35,671 to 50,359)	59,248 (46,609 to 70,675)	17,740 (14,680 to 20,089)
Prevented cardiovascular disease cases	9,675 (7,080 to 12,213)	14,060 (9,727 to 18,179)	10,177 (7,408 to 13,040)	11,695 (8,029 to 15,247)	18,595 (11,264 to 25,721)	4,567 (4,190 to 4,949)
Per capita change in BMI, kg m^−^^2^	−1.53 (−1.54 to −1.52)	−1.96 (−1.97 to −1.95)	−1.47 (−1.48 to −1.46)	−1.59 (−1.6 to −1.58)	−2.09 (−2.1 to −2.07)	−0.96 (−0.96 to −0.95)
Prevented all-cause mortality	1,236 (932 to 1,496)	1,741 (1,247 to 2,170)	1,225 (904 to 1,521)	1,456 (1,042 to 1,799)	2,240 (1,729 to 2,726)	552 (475 to 610)

^a^Pathways: ‘CCC 2030’, a 20% reduction in all meat and dairy; ‘CCC 2050’, a 35% reduction in all meat and 20% reduction in all dairy; ‘Scottish Dietary Goal (red meat 70 g per day max)’, reducing red meat to a maximum intake of 70 g per day and 20% reduction in all dairy; ‘Red meat 60 g per day max’, reducing red meat to a maximum intake of 60 g per day and 20% reduction in all dairy; and ‘Red meat 31 g per day max’, reducing red meat to a maximum intake of 31 g per day and 20% reduction in all dairy.

In the most effective pathway, over 10 years, there were 59,248 (95% UI, 46,609 to 70,675) prevented type 2 diabetes cases (~25.5% of annual cases), 18,595 (11,264 to 25,721) prevented cardiovascular disease cases (~5.8% of annual cases), a population average change in body mass index (BMI) of −2.09 kg m^−^^2^ (−2.10 to −2.07 kg m^−^^2^) and 2,240 (1,729 to 2,726) prevented all-cause mortalities (~3.9% of annual deaths) (Table 2). Compared with an equivalent pathway assuming isocaloric substitution, 66% of the prevented type 2 diabetes cases were a result of weight loss (Supplementary Table 12).

### Environmental outcomes

**Fig. 3:**
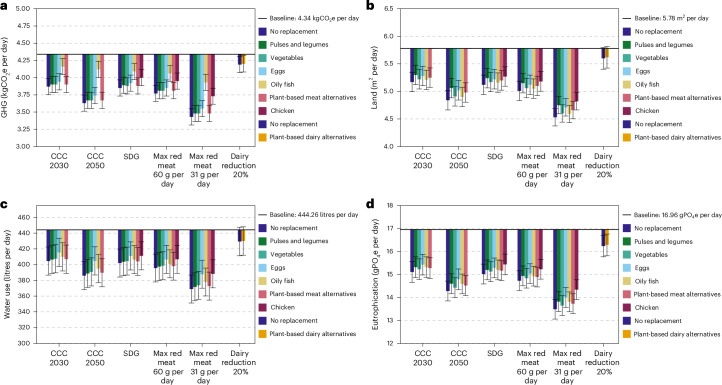
Impact on environmental impacts in each pathway. **a**–**d**, Impact on per capita GHG emissions (kgCO_2_e per day) (**a**), land use (m^2^ per day) (**b**), water use (litres per day) (**c**) and eutrophication (gPO_4_e per day) (**d**) in each meat and dairy reduction pathway in a representative sample of adults 16+ years living in Scotland (*n* = 3,447). Each bar represents the difference between the mean per capita impacts at baseline and each pathway. Error bars represent 95% UIs, calculated as 1.96 multiplied by the standard error of the difference in impact at baseline and each pathway. GHG, GHG emissions.

All pathways resulted in a significant net reduction in GHG emissions, with pathways involving the reduction of red meat in high consumers having slightly greater reductions in dietary GHG emissions relative to the equivalent CCC pathway (Table 2). For example, the CCC 2030 pathway with no replacement decreased GHG emissions by 0.47 kgCO_2_e per capita per day (95% UI, −0.59 to −0.35 kgCO_2_e per capita per day) whereas achieving the same overall reduction in all meat by reducing red meat in high consumers (red meat 60 g per day max pathway) resulted in a decrease of 0.57 kgCO_2_e per capita (including non-consumers) per day (−0.69 to −0.45 kgCO_2_e per capita per day). A similar pattern was observed for other environmental outcomes. All meat and dairy replacement pathways resulted in significant net reductions in all environmental outcomes (Fig. 3a–d).

### Diet costs

**Fig. 4:**
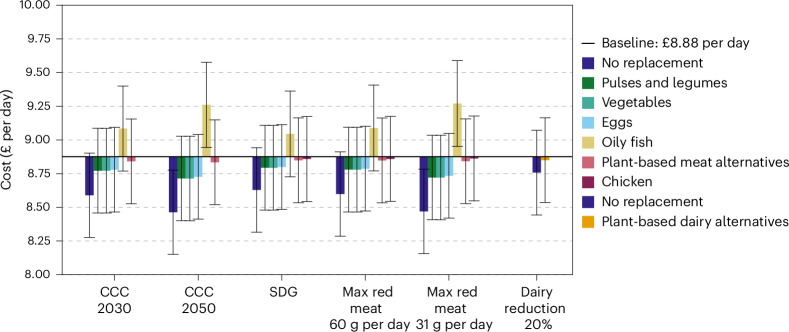
Impact on diet cost in each pathway. Impact on the per capita cost of diets in each meat and dairy reduction pathway in a representative sample of adults 16+ years living in Scotland (*n* = 3,447). Each bar represents the difference between the mean per capita diet costs at baseline and each pathway. Error bars represent 95% UIs, calculated as 1.96 multiplied by the standard error of the difference in cost at baseline and each pathway.

The average cost decreased by £0.41 per day (95% UI, −£0.72 to −£0.09) in the most effective pathway (Fig. 4 and Table 2) and by £0.41 per day (95% UI, −£0.72 to −£0.10) in the equivalent CCC pathway (pathway 7). Replacement with vegetables or pulses and legumes retained the greatest average cost savings in the most effective pathway, with both resulting in an average cost saving of £0.15 per day (95% UI, −£0.16 to £0.46). Replacement with oily fish in this pathway and the equivalent CCC pathway (pathway 11) resulted in the only statistically significant increase in cost, with an increase of £0.39 per day (95% UI, £0.08 to £0.71) and £0.38 per day (95% UI, £0.07 to £0.70) respectively. There was no significant difference in cost savings between the most and least deprived quintiles in the most effective pathway, at £0.40 per day (95% UI, −£0.59 to £1.39) and £0.38 per day (95% UI, −£0.13 to £0.90) respectively. Demographic differences for other outcomes in the most effective pathway are provided in Table 3 and for all pathways in Supplementary Data.

**Table 3:** Mean per capita impacts (95% UI^a^) on nutrient intake (zinc, calcium, iodine and selenium), the environment (GHG emissions), cost of diets and chronic disease incidence (cardiovascular disease) in the most effective pathway, reducing red meat intake to a maximum of 31 g per day together with a 20% reduction in all dairy with no replacement across population subgroups in a representative sample of adults 16+ years living in Scotland

	Zinc, mg per day	Calcium, mg per day	Iodine, µg per day	Selenium, µg per day	Dietary GHG emissions, kgCO_2_e per day	Cost of diets, £ per day	Cardiovascular disease, prevented cases over 10 years
Men	−1.48 (−1.81 to −1.15)	−94.18 (−141.69 to −46.67)	−18.00 (−28.84 to −7.16)	−4.59 (−7.12 to −2.06)	−1.1 (−1.28 to −0.92)	−0.48 (−0.99 to 0.04)	12,178 (7,300 to 16,712)
Women	−1.0 (−1.24 to −0.76)	−84.34 (−109.58 to −59.1)	−15.98 (−21.66 to −10.30)	−2.89 (−4.56 to −1.22)	−0.73 (−0.87 to −0.59)	−0.34 (−0.64 to −0.04)	6,417 (3,963 to 8,998)
16–24 years	−1.17 (−2.19 to −0.15)	−84.33 (−198.11 to 29.45)	−15.56 (−32.98 to 1.86)	−3.83 (−9.71 to 2.05)	−0.96 (−1.43 to −0.49)	−0.42 (−1.57 to 0.73)	62 (35 to 87)
25–34 years	−1.1 (−1.57 to −0.63)	−78.09 (−136.13 to −20.05)	−14.14 (−26.25 to −2.03)	−3.52 (−7.2 to 0.16)	−0.8 (−1.11 to −0.49)	−0.36 (−1.08 to 0.36)	258 (147 to 372)
35–44 years	−1.37 (−1.9 to −0.84)	−95.82 (−158.5 to −33.14)	−17.55 (−32.98 to −2.12)	−4.08 (−7.57 to −0.59)	−0.98 (−1.27 to −0.69)	−0.46 (−1.13 to 0.22)	1,044 (584 to 1,500)
45–54 years	−1.22 (−1.73 to −0.71)	−88.05 (−147.18 to −28.92)	−16.39 (−28.48 to −4.3)	−3.5 (−6.66 to −0.34)	−0.91 (−1.2 to −0.62)	−0.38 (−1.10 to 0.34)	2,457 (1,394 to 3,451)
55–64 years	−1.29 (−1.66 to −0.92)	−91.53 (−139.57 to −43.49)	−17.76 (−28.29 to −7.23)	−3.85 (−6.61 to −1.09)	−0.93 (−1.17 to −0.69)	−0.41 (−0.96 to 0.14)	4,496 (2,600 to 6,380)
65–74 years	−1.3 (−1.69 to −0.91)	−94.28 (−144.89 to −43.67)	−19.11 (−33.22 to −5.0)	−3.71 (−6.49 to −0.93)	−0.94 (−1.19 to −0.69)	−0.42 (−0.91 to 0.06)	5,631 (3,647 to 7,403)
75+ years	−1.16 (−1.63 to −0.69)	−93.46 (−165.24 to −21.68)	−19.11 (−38.61 to 0.39)	−3.44 (−7.36 to 0.48)	−0.85 (−1.2 to −0.5)	−0.39 (−1.13 to 0.35)	4,644 (2,853 to 6,487)
SIMD 1 (most deprived)	−1.28 (−1.85 to −0.71)	−80.43 (−140.6 to −20.26)	−15.41 (−30.44 to −0.38)	−4.2 (−9.55 to 1.15)	−1.03 (−1.32 to −0.74)	−0.40 (−1.39 to 0.59)	2,620 (1,469 to 3,772)
SIMD 2	−1.29 (−1.64 to −0.94)	−87.45 (−127.26 to −47.64)	−17.43 (−27.31 to −7.55)	−3.66 (−6.17 to −1.15)	−0.93 (−1.18 to −0.68)	−0.41 (−1.04 to 0.23)	4,272 (2,495 to 5,966)
SIMD 3	−1.23 (−1.64 to −0.82)	−90.99 (−142.38 to −39.6)	−17.13 (−28.6 to −5.66)	−3.84 (−6.88 to −0.8)	−0.89 (−1.2 to −0.58)	−0.42 (−0.93 to 0.09)	4,895 (3,106 to 6,664)
SIMD 4	−1.22 (−1.67 to −0.77)	−89.09 (−137.8 to −40.38)	−16.83 (−27.16 to −6.5)	−3.56 (−5.87 to −1.25)	−0.92 (−1.17 to −0.67)	−0.42 (−0.98 to 0.14)	2,955 (1,639 to 4,283)
SIMD 5 (least deprived)	−1.15 (−1.56 to −0.74)	−96.52 (−144.01 to −49.03)	−17.78 (−31.17 to −4.39)	−3.37 (−6.19 to −0.55)	−0.79 (−1.03 to −0.55)	−0.38 (−0.90 to 0.14)	3,851 (2,553 to 5,144)

^a^The 95% UIs on nutrient intake, GHG emissions and cost of diets calculated as ±1.96 multiplied by the standard error of the indicator per capita. The 95% UIs on prevented chronic disease cases are estimated by taking the bottom 2.5% and top 97.5% of the distribution of prevented cases.

## Discussion

We found that the CCC recommendations to reduce overall meat consumption by 20% by 2030, rising to 35% by 2050^2^, can be achieved in Scotland by reducing red meat consumption in high consumers to a maximum of 60 g per day and 31 g per day, respectively. Results of our nationally representative simulation suggest that such an emphasis on red meat consumption in high consumers of red meat would prevent more chronic disease cases, produce slightly greater reductions in environmental harms—not just GHG emissions but also land and water use, as well as eutrophication—and have comparable cost savings. Currently, meat and meat products in Scotland account for a small proportion of the cost of diets and nutrient intake relative to their contribution to environmental harms. Dairy, meanwhile, accounts for a smaller portion of the cost of diets and environmental harms than meat and is a substantial source of key nutrients such as calcium and iodine. Thus, reducing dairy consumption would have a smaller impact on environmental harms and food expenditure than reducing red meat consumption, while carrying a greater risk of introducing or exacerbating nutrient deficiencies in certain population groups based on current Scottish dietary patterns. Gram-for-gram replacements of meat and dairy with nutrient rich substitutes ranging from vegetables to beans to eggs can attenuate effects on nutrient intake, improve adherence to dietary guidance for fruit and vegetables, and provide environment benefits with little impact on diet costs.

We found that total GHG emissions associated with food consumed by adults living in Scotland—estimated in our analysis to be 10.4 MtCO_2_e—exceed those of the agricultural sector in Scotland (7.7 MtCO_2_e)^21^, further supporting the need for a change in food consumption patterns alongside changes in production practices. This discrepancy arises in part from Scotland’s reliance on food imports, particularly pork, chicken, white fish and out-of-season fruits and vegetables^22^. The most comprehensive analysis of GHG emissions from food consumed in the UK, including imported food, estimated emissions of 154.8 MtCO_2_e in 2020^23^. Assuming emissions are proportional to population, this would amount to 12.7 MtCO_2_e per year in Scotland. The difference is probably due to the inclusion of packaging, transport, waste disposal and energy use by food retailers, hospitality, food service and households^23^, which were excluded from the life cycle assessment database in our analysis^15,16^. Together, these stages of the food supply chain accounted for 24% of total emissions^23^; not accounting for these would amount to about 9.6 MtCO_2_e per year, which is similar to our estimate.

The cost savings from the most effective pathway were £0.40 per day for those living in the most deprived areas of Scotland and £0.38 per day for those in the least deprived when meat and dairy were not replaced. When replaced with vegetables or legumes, the average cost savings were £0.15 per day in both pathways. These results are comparable to a previous diet optimization study that found an average saving of £0.21 per day^14^. By contrast, that study found greater cost savings in higher-income households (£0.47 per day) as compared with lower-income households (£0.23 per day)^14^, while we did not find a statistically significant difference in cost savings between the most deprived quintile and the least deprived quintile (Supplementary Data).

While current UK dietary guidelines recommend limiting red meat consumption to no more than 70 g per day^24^, this remains considerably higher than recommendations in other countries that also account for environmental impacts such as the Nordic Nutrition recommendations of 50 g per day^25^. Our results suggest that reducing red meat consumption to no more than 60 g per day will meet the 2030 CCC recommendation and provide benefits to population health and the environment while having little impact on food expenditure or nutrient intake.

Self-reported dietary intake is typically underreported, with the most recent doubly labelled water substudy of the UK National Diet and Nutrition Survey finding energy intake to be underreported by 33–41%^26^. Absolute values for nutrient intake, environmental outcomes and cost of diets should therefore be considered lower bounds (that is, they are conservative estimates). As the change in these outcomes is not affected by baseline intake, we focus on the change relative to baseline for each pathway as the primary results. We did not estimate the proportion of the population with nutrient intakes below the dietary reference values, as these proportions are likely to be overestimated when energy and, hence, nutrient intakes are underreported. Our results reflect impacts on nutrient intake and do not take into account bioavailability, which is influenced by many factors not measured in SHeS such as an individual’s micronutrient status and genetics. Future research could explore how the bioavailability of iron and zinc may vary under different meat and dairy reduction pathways. The choice of substitutes was largely guided by existing dietary goals in Scotland, for example, to increase the consumption of vegetables and oily fish. However, future work could explore other nutrient-rich substitutes, such as mussels or nuts. Furthermore, the relative risks used for estimating impacts on chronic diseases were from meta-analyses that aimed to estimate the independent impact of unprocessed and processed red meat consumption^27,28^ or dairy consumption^29^. We did not estimate the impacts of targeted replacements of meat and dairy on chronic disease incidence because dose–response relative risk associations for targeted replacements of meat and dairy are scarce. However, we performed a supplementary analysis to determine the relative impact of caloric reduction on disease prevention by comparing the prevented cases with simulations assuming isocaloric substitution, finding that the caloric reductions were the primary contributor to prevented cases for most health outcomes (Supplementary Tables 11 and 12). Our estimates for prevented all-cause mortalities are probably conservative due to the association between unprocessed and processed red meat intake and the risk of other health outcomes such as several cancers^4,30^.

Adopting the CCC recommendations to reduce both meat and dairy is likely to have considerable benefits in terms of environmental impacts and public health, with limited impact on nutrient intake or household budgets, particularly if red meat is replaced with plant-based alternatives or eggs. Regulations to ensure consistent fortification of plant-based dairy alternatives with calcium and iodine should be considered given that milk and milk products are an important source of these nutrients together with the heterogeneity in nutrient content of plant-based dairy alternatives available in the UK^18,31^. As a next step, work is needed to understand what levers are available at the local and national level to reduce red meat consumption while simultaneously promoting the consumption of healthy alternatives^32^. The Good Food Nation (Scotland) Act 2022^33^ and forthcoming good food nation plans represent an opportunity to adopt measures to accelerate progress towards the CCC dietary recommendations and Scottish Dietary Goals. Current rates of decline in red meat consumption across the UK are not sufficient to meet a 20% decline in all meat by 2030^34^. Policies such as the sugary drinks levy have proven to be effective at shifting population intakes in the UK^35^ and could be considered in the context of red meat. Reducing the price of plant-based meat alternatives along with regulations to ensure they are nutritionally appropriate substitutes could also facilitate behaviour change^19,36^. Additional fiscal measures could include value-added tax (VAT) reform by increasing VAT on meat and dairy products (meat and dairy are currently VAT exempt^37^) while subsidizing alternatives^38^. Future work could incorporate demand system modelling to assess the impact of price changes, including those due to fiscal reform or following geopolitical or environmental change.

Given that consumption of red meat in the Scottish context is highest on Sundays, red meat is largely purchased at supermarkets and beef dishes and sandwiches are the main contributors among high consumers^19^, modifying traditional meat-centric dishes and sandwiches could be an effective place to start. Adopting the CCC recommendations is a necessary first step, but achieving population-level change will require policies that make healthy plant-based foods widely available and a more desirable, convenient and affordable choice than red meat.

## Methods

### Nutrient intake

The average daily nutrient intake of all respondents that completed at least 1 day of dietary recall (*n* = 3,447) from the 2021 round of SHeS was computed to determine baseline intake. Most of the sample (88.3%) provided 2 days of dietary recall data. We evaluated intake of all 54 nutrients included in the UK nutrient databank^39^. Nutritional supplements were excluded from the analysis. The meat and dairy content of all food items were obtained using the Food Standards Agency Recipe database^40^ and the UK nutrient databank^39^, which contains both the ingredients in each reported food item and the nutrient content per 100 g of both the total food item and each respective ingredient^39^. Data on the grams of each dairy food group in composite food items were not available in the UK nutrient databank^39^; these data were obtained from a separate analysis, published elsewhere^41^.

The 20% reduction in dairy included reductions of both non-composite dairy items (for example, milk) and items that included dairy as an ingredient (for example, lasagne). For non-composite items, the gram weight of the item was reduced by 20%, while for composite items, the 20% reduction was applied to gram weight of each dairy ingredient. Similarly, in the CCC pathways, the total gram weight of all non-composite meat items, as well as the meat content of composite items, including white meat, was reduced by 20% and 35%, respectively. In pathways where red meat intake was reduced among high consumers to meet a maximum daily intake, a sampling approach was employed to account for the different impacts on all indicators from meeting the maximum daily intake via reductions in different meat food types (Supplementary Information pp. 3, 4). As there was no a priori reason to favour a reduction in one red meat food group over another, we randomly sampled from combinations of reductions of each red meat food group in both composite and non-composite foods consumed by individuals above the maximum threshold (to either 70 g per day, 60 g per day or 31 g per day) in 10-g increments, plus the remainder, until the maximum daily intake was reached exactly. We ran a sensitivity analysis to ensure the results were robust to the choice of increment size by comparing the maximum absolute difference in intake across red meat food groups in the most effective pathway with an increment size of 10 g to an increment size of 1 g, finding that the average maximum difference was 1.5 g (Supplementary Information p. 4). The associated gram weight reduction of non-composite meat items was then used to compute the impact on nutrient intake. For composite meat items, the gram weight of each meat ingredient was reduced incrementally until the maximum intake threshold was reached. This approach assumes that each meat consumer is equally likely to reduce one meat food group as another. The gram weight of the reduction of each meat and dairy ingredient was then used to calculate the associated reduction in nutrient intake using the nutrient content per gram, taken from the UK nutrient databank^39^.

We considered gram-for-gram replacement pathways for the red meat reduction: pulses and legumes, vegetables, non-smoked oily fish, eggs, plant-based meat alternatives and, for red meat pathways, gram-for-gram replacement with poultry. With dairy, we considered gram-for-gram replacements with plant-based milks, plant-based yoghurt and plant-based solid fats. Cheese and cream were not replaced in any pathway given limited availability in the Scottish food retail environment^18^. The frequency of reported foods in each substituted food group in SHeS 2021 was used to derive a weighted composite substitution (Supplementary Information pp. 6–13 and Supplementary Data). For example, 50% of the reported food items in the oily fish food category consisted of the item ‘Salmon, grilled or oven baked’. As such, 50% of the nutrients in the oily fish substitute consisted of the nutrient content of this item. In each pathway we computed both the absolute level of nutrient intake for each nutrient and the change in the nutrient intake compared with baseline, in the overall population and in population subgroups (sex, age group and SIMD).

### Environmental outcomes and diet cost

For each pathway, the impact on four environmental indicators was assessed: GHG emissions, land use, water use and eutrophication, along with the cost of diets. As environmental impact data were not available in SHeS 2021, these data were obtained by mapping items in the UK nutrient databank to appropriate matches in foodDB^15,16^. foodDB contains standardized environmental impacts and price data per 100 g of approximately 70,000 items available in UK-based supermarkets, derived by combining data from a large meta-analysis of life cycle assessments with an ingredient decomposition analysis^16,42^. The stages in the life cycle assessments include agricultural production and the processing and transport of agricultural commodities but do not include post-production processing, packaging or the transport of processed commodities to retail stores. For each of the environmental impacts, each food in foodDB has a distribution of possible measures based on Monte Carlo analyses where region and production method for the ingredients are allowed to vary. We use the median value from these distributions for our point estimates. The environmental impact and cost of each UK nutrient databank item per 100 g was calculated by averaging the median impacts and costs of all foodDB items that were matched to each item in the UK nutrient databank. Each of the matches between the UK nutrient databank and foodDB items was manually verified by members of the research team. The total environmental impact and diet costs were calculated by multiplying the environmental impact and cost per gram by the gram weight of each consumed item, before summing over all items and dividing by the number of days of recall. The subsequent impact in each simulation pathway followed the same approach as estimating the change in nutrient intake. To estimate the population-level environmental impacts after adjusting for underreporting, we calculated the impact per calorie for each individual based on the self-reported data and multiplied this quantity by the reported gap in calories, based on their age and sex (Supplementary Information p. 18). This was then added to their individual impact based on self-reported data, before summing these adjusted impacts over the entire population to obtain the final estimates for population-level environmental impact.

### Chronic disease, mortality and obesity

Data on demographics, health status and dietary intake were used as input to a previously developed microsimulation model, which was adapted to SHeS 2021 to estimate prevented cases of type 2 diabetes, cardiovascular disease, all-cause mortality and population average BMI^43^. In the case of type 2 diabetes and cardiovascular disease, pre-existing disease risk models^44,45^ were used to predict baseline disease risk in the absence of dietary data, before being multiplied by a relative risk associated with their unprocessed red meat intake, processed red meat intake and total dairy intake taken from meta-analyses in the case of diabetes^27,29^ and a large-cohort study for cardiovascular disease^28^. Incidence was then estimated by multiplying each individual’s disease risk by their sample weight scaled to be representative of the population size, before summing the results over all respondents. In instances where the incidence estimates did not match the observed incidence in a demographic group, the risk was calibrated to match that observed in the first simulation year. At the end of each simulation year, disease risk factors such as age and BMI were updated to calculate the updated risk for subsequent years. The dietary change was assumed to happen in the first year and remain fixed for the following 10 years.

Mortality risk was not directly related to dietary risk factors, but rather related indirectly via change in disease status, which in turn influenced mortality risk. Baseline mortality probabilities were estimated on the basis of age and sex from 2019 national mortality statistics^46^, before being multiplied by a relative risk of all-cause mortality should the individual have a chronic disease^47,48^. The number of mortalities associated with that disease was then calculated as the original sample weight multiplied by the mortality risk, with the result then being subtracted from the original sample weight, as described elsewhere^43^.

To estimate the impact of the reduction in meat and dairy intake on obesity, we used a pre-existing model^49^ that estimates the change in body weight following a change in energy intake, accounting for other variables such as sex, resting metabolic rate and physical activity levels. The decrease in weight is then used to estimate a decrease in BMI when combined with height data in SHeS 2021 (Supplementary Information pp. 27, 28).

### Uncertainty analysis

Uncertainty estimates on the health outcomes were obtained by sampling from the distributions of known sources of uncertainty in each of the 50 iterations of each pathway. Fifty iterations were chosen because the width of the 95% UIs for the health outcomes did not decrease beyond 40 iterations (Supplementary Information p. 44). Sources of uncertainty included the relative risk relations between unprocessed red meat, processed red meat, total dairy intake and chronic disease risk, the parameter posterior distributions for the systolic blood pressure, high-density lipoprotein cholesterol, total cholesterol, height and weight imputation models, and the metabolic equivalent scores for different physical activity levels. The uncertainty in nutrient intakes, environmental outcomes and costs accounts for both the survey design and, for the red meat pathways, 50 Monte Carlo iterations in which different combinations of red meat food groups (for example, beef versus sausages) were reduced to a specified maximum intake level. The uncertainty in the environmental impacts also account for the within-item uncertainty in the matching to foodDB, estimated by taking the standard deviation of the medians of each matched foodDB item. More details on the uncertainty analysis are provided in Supplementary Information pp. 18–20 and 38–44.

### Reporting summary

Further information on research design is available in the Nature Portfolio Reporting Summary linked to this article.

## Supplementary information

Supplementary Information Single pdf containing Supplementary Discussion, Tables 1–12 and Figs. 1–19.

Reporting Summary

Supplementary Data Relative contribution of each main food category in SHeS 2021 to nutrient intake, environmental impact and cost. SHeS, Scottish Health Survey. Mapping between meat containing items and the associated meat ingredient for each meat food group in the simulation. Breakdown of the food items in each of the meat and dairy substitute composite food groups. Estimated changes and absolute values of per capita nutrient intake, per capita cost, per capita environmental impacts and prevented cases of each health outcome for all relevant pathways in the entire sample, by sex, age group and by SIMD quintile.

## Data Availability

The Scottish Health Survey 2021 data can be obtained from the UK Data Service (https://ukdataservice.ac.uk/, 10.5255/UKDA-SN-9048-2). Due to legal constraints, product-level data in foodDB and the mapping between the nutrient databank and foodDB are not publicly available. Access for the purpose of replication can be requested from trisha.gordon@ndph.ox.ac.uk. Data on the estimated environmental impacts and costs for food items in the UK Nutrient Databank derived from foodDB are available via Edinburgh DataShare^50^.

## Code availability

All source code is available via Zenodo at 10.5281/zenodo.20447123 (ref. ^51^), with any subsequent updates available via GitHub at https://github.com/jsfken/mSHIFT_SHeS.
