# Supplementary material for: Reduced meat and dairy consumption improves health, environmental and most nutritional outcomes without increasing diet costs among Scottish adults
Source: Nat Food. 2026 Jul 3;7(7):711–21. doi: 10.1038/s43016-026-01384-3 (PMC13388101; doi:10.1038/s43016-026-01384-3)
Supplement: Supplementary file 1 — Single pdf containing Supplementary Discussion, Tables 1–12 and Figs. 1–19. [file 43016_2026_1384_MOESM1_ESM.pdf]

nature food

Supplementary information https://doi.org/10.1038/s43016-026-01384-3

Reduced meat and dairy consumption improves health, environmental and most nutritional outcomes without increasing diet costs among Scottish adults

In the format provided by the authors and unedited

Supplementary material

Joe Kennedy, Michael Clark, Cristina Stewart, Ricki Runions, Alexander Vonderschmidt, Sarah M. Frank, Peter Scarborough, Fiona Comrie, Alana McDonald, Geraldine McNeill, Peter Alexander, Lindsay M. Jaacks

Contents

Modelling the impact on nutrient intake 2
- Diet and nutrient data sources 2
- Deriving intake thresholds for high consumers 2
- Meat and dairy reduction modelling 3
- Modelling the impact of meat and dairy substitutions 6

Modelling the impact on environmental outcomes and cost of diets 14
Environmental impact adjusted for usual dietary intake 17
Uncertainty estimation 18
Food category contributions to baseline impacts, cost and nutrient intake 21
Modelling the impact on chronic diseases, mortality and obesity 21
References 47

Modelling the impact on nutrient intake

Diet and nutrient data sources

Dietary data were from the 2021 Scottish Health Survey (SHeS) [1]. All respondents (n=3,447) reported the food and drink items consumed across either one day or two days of recall, with most of the sample (88.3%) providing two days of recall. The nutrient content of all reported items in SHeS are calculated from data in the UK Nutrient Databank (NDB) [2], a standardised dataset with estimates for the nutrient content per 100g of each item across 54 macro and micronutrients. Baseline intake of each nutrient per individual was calculated by summing up the nutrient content in all reported items and dividing by the number of days of recall for that individual.

In addition to the nutrient content, estimates of the disaggregated gram weight of different foods per 100g were also provided in the NDB [3]. Disaggregated meat in the NDB includes "Beef", "Lamb", "Pork", "Processed red meat", "Other red meat", "Burgers", "Sausages", "Offal", "Poultry", "Processed poultry" and "Game birds". Unprocessed red meat was defined as "Beef", "Pork", "Lamb", "Burgers" and "Other red meat". Processed red meat was defined as "Processed red meat", "Sausages" and "Offal". In the pathways CCC 2030 and CCC 2050, we also applied reductions to the food groups "Poultry", "Processed poultry" and "Game birds". Baseline daily intake of each meat type was calculated in the same manner as for nutrients, by summing the gram weight of the meat type consumed in every reported item and dividing by the number of days of recall. Daily consumption of all meat, unprocessed red meat and processed red meat was then calculated by summing the daily intakes of each of the associated meat types. There were three poultry containing items in the "Sausages" and "Offal" food groups: "Chicken and vegetable soup, homemade", "Chicken liver" and "Chicken/turkey sausage". In estimating baseline red and red processed meat intake, the contribution from these three poultry containing items to "Sausages" and "Offal" intake were excluded.

Disaggregated dairy was not available in the NDB. In this instance, we used data from a separate analysis[4] to estimate the gram weight per 100g of "Milk skimmed", "Milk, semi-skimmed", "Milk Whole", "Cream skimmed", "Cream whole", "Cheese skimmed", "Cheese semi-skimmed", "Cheese whole", "Yoghurt skimmed", "Yoghurt semi-skimmed", "Yoghurt whole" and "Butter".

Deriving intake thresholds for high consumers

Once the daily consumption of red and red processed meat was calculated it was possible to calculate the percentage reduction in all meat (including "Poultry", "Processed poultry" and "Game birds") that corresponds to a chosen maximum daily intake threshold (herein maximum threshold) of red and red processed meat (herein red meat) as follows. If an individual consumed greater than the specified maximum threshold, then the required reduction was calculated as the difference between their current red meat intake and the maximum threshold. After multiplying by the sample weights and summing over the entire population, we derived the total gram weight of the red meat reduction for a given maximum threshold. By taking the ratio between this red meat reduction and the sample weighted sum

of the consumption of all meat across all individuals we can derive the percentage level reduction in all meat that corresponds to different maximum thresholds. In **Figure S1** we show this relationship for different values of the maximum thresholds. From this relationship, we derived the maximum intake of red meat to achieve both of the CCC meat reduction pathways—60g/day for CCC 2030 and 31g/day for CCC 2050—as well as the percent reduction in all meat should the current Scottish Dietary goal of 70g per day of red meat be met, namely 16%.

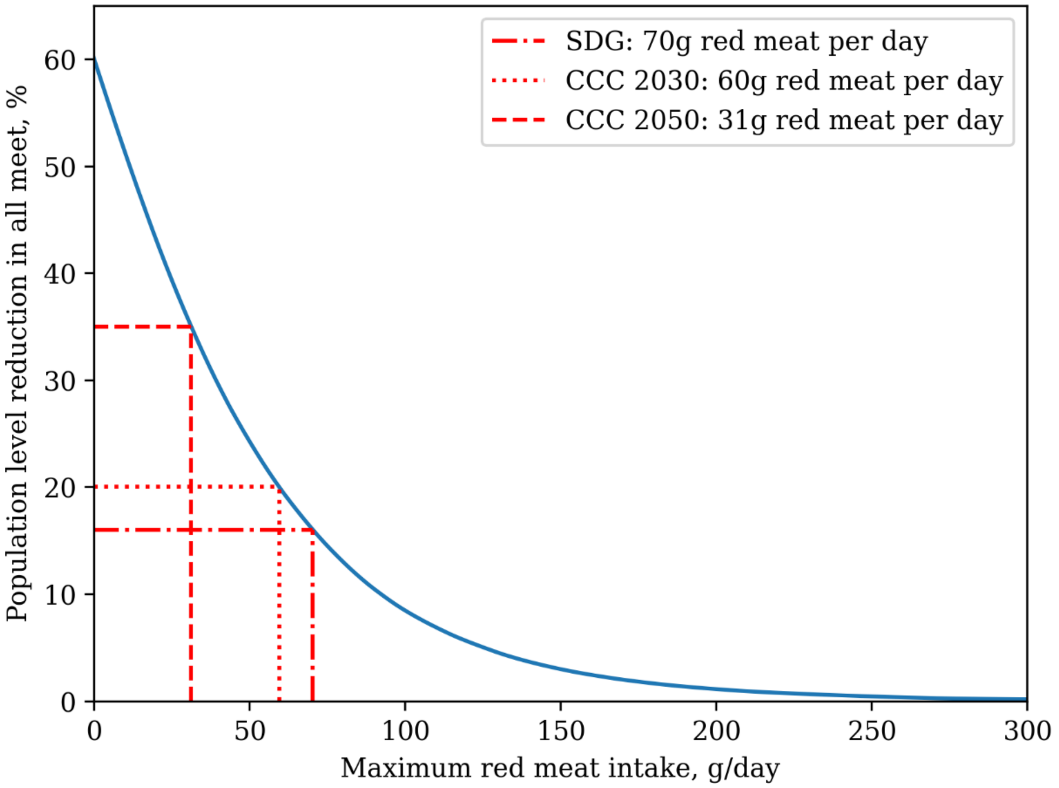

Figure S 1. Relation between the maximum daily intake of red meat with the associated percentage reduction in all meat. The three reduction pathways are highlighted to demonstrate how the percentage reduction in all meat leads to each maximum daily intake threshold set in the simulation pathways. Data are based on average daily intakes from the 2021 Scottish health Survey (n=3,447). CCC: Climate Change Committee, SDG: Scottish Dietary Goal.

Meat and dairy reduction modelling

In all simulation pathways, reductions in meat and dairy were applied to each of the disaggregated meat and dairy types. In pathways CCC 2030 and CCC 2050, percent level reductions were applied to each meat and dairy type regardless of consumption level. These reductions were applied to the meat and dairy ingredients in composite items (e.g. “Beef lasagne”) and non-composite meat and dairy items (e.g. “Minced beef”). For example, in pathway CCC 2030, which required a 20% reduction in all meat, if the disaggregated pork content in a reported item was 100g, then the reduction in pork consumption associated with that item would be 20g. If the same food item also contained 50g of poultry, then in pathways CCC 2030 and CCC 2050, the reduction in poultry consumption associated with that item was 10g.

In the pathways focusing on high red meat consumers, meat reductions were applied to the disaggregated red meat types such that the total red meat intake averaged across both days of recall did not exceed a specified maximum intake level. For example, if the maximum intake was 70g red meat per day, then an individual with two days of recall who consumed greater than 70g per day when averaged across both days, could not consume more than 140g of red meat after summing across both days of recall. As there are multiple pathways to reach this maximum intake for high red meat consumers depending on what disaggregated red meat types to reduce, an iterative process was applied to each high consumer. The first step was to randomly select a disaggregated red meat type among all disaggregated red meat types that were consumed and reduce the intake of that disaggregated red meat type by 10g. This process was then repeated until the individual's total intake of red meat was less than 10g away from the maximum intake. Finally, the remaining reduction was applied to a randomly selected disaggregated red meat type if the intake of that disaggregated red meat type was greater than the remainder. For example, consider an individual who consumed 100g of beef, 50g of pork and 64g of sausages across two days of recall and a maximum threshold of 70g. In total, this individual consumed 214g of red meat and so to meet the pathway requirements they need to reduce their intake by 74g. After randomly sampling the beef, pork and sausages disaggregated red meat types and applying the reductions in 10g increments, one possible realisation is a reduction in beef of 30g, a reduction in pork of 30g and a reduction in sausages of 10g. The additional 4g required to meet the threshold is then randomly added to one of these disaggregated red meat types. For example, after applying the reductions, the individual's new intake is 34g of beef, 30g of pork and 10g of sausages. This process was then repeated for 50 iterations to ensure that the variation in the impact of each outcome across nutrient intake, health, diet costs and the environment, also accounts for the variable impact of reducing different disaggregated red meat types to meet the maximum threshold. In **Table S1** we provide the sample weighted percentages of the number of respondents who consumed above each maximum threshold considered in this study. To ensure the choice of increment size (e.g. 10g) had a marginal impact on the results, we ran a sensitivity analysis by comparing the average reduction in each red meat food group across all reduction combinations for a maximum intake of 31g with two methods: 10g increments with random allocation of the remainder and 1g increments. The average of the maximum absolute difference across all red meat food groups between the two methods among those that reduced their consumption was 1.5g, well within the typical individual level variation in self-reported dietary recall[5] and negligible when compared to the error associated with mis-reporting.

Table S1. Sample weighted percentage of respondents in SHeS 2021 that consumed more than the three maximum thresholds of daily red meat intake examined in this study.

Maximum daily red meat intake	% sample above threshold
70g	27.6%
60g	32.8%
31g	53.7%

A 20% dairy reduction was then applied in these pathways regardless of consumption level (i.e. to all consumers).

Once the gram weight of the reductions in disaggregated meat and dairy types were derived for each respondent, the impact on nutrient intake for all 54 nutrients was derived. For non-composite meat and dairy items, the new nutrient intake levels were calculated by

multiplying the nutrients per gram of the entire item by the gram weight of the reduction and subtracting this value from the baseline nutrient intake. The new nutrient intake level was then calculated by subtracting this reduction from their baseline nutrient intake. For example, a respondent who reported consuming 100g of semi-skimmed milk would then consume 80g after applying the 20% dairy reduction, with the change in nutrient intake derived from subtracting the nutrient content of 20g of semi-skimmed milk for each nutrient.

For composite meat and dairy items, the nutrients of the meat and dairy ingredients were subtracted from their total nutrient intake, rather than the nutrient content of the entire item. In the case of all disaggregated meat types other than offal, each meat containing item was assigned a non-composite meat item to each meat group the item contained. These meat ingredients were chosen from all non-composite meat items in SHeS with the assigned ingredients verified independently by two members of the research team. For example, the food item "Chilli con carne" contains beef, with the non-composite beef food item chosen as "Minced beef, stewed". Therefore, if a respondent reported consuming "Chilli con carne" with 100g of beef and the final beef reduction for that item in a particular pathway was 30g, then the nutrients in 30g of "Minced beef, stewed" were subtracted from the total nutrient content of the entire item. A single meat ingredient was assigned to each meat food group for each meat containing item. In the case of offal items, the nutrient content of the entire food item was reduced under the assumption that offal meat was unlikely to be consumed in the absence of the composite ingredients.

We provide the full mapping of meat ingredients assigned to meat containing items in the **Supplementary data**. Due to this matching, we found that for some individuals who had a particularly low baseline nutrient intake for certain nutrients, after applying the reductions, the nutrient intake became negative due to differences in the nutrient content of the matched meat item and that used to derive the original nutrient content of the meat containing item. For example, the meat containing item "Beef burger/hamburger, in a bun, not quarter pounder" does not contain vitamin D, while the matched non-composite meat item "Beef burger, grilled (no bun)" contained 0.005mg of vitamin D per gram. In instances of negative nutrient intake, intake was manually set to zero.

For composite dairy items, the Food Standards Agency (FSA) recipe database[6] was used to assess the nutrient contribution of each dairy ingredient in each food type. In instances where there were multiple dairy ingredients in a single dairy food type, the reduction was applied in proportion to the contribution of each dairy ingredient to gram weight of the total gram weight of the food type in the item. For example, 100g of the item "Milkshake ready to drink (e.g. Frijj, yazoo)" with food code 8215 contains the following dairy ingredients in the FSA recipe database:

- "MILK SEMI-SKIMMED PASTEURISED SUMMER", 46.65g
- "MILK SEMI-SKIMMED PASTEURISED WINTER", 46.5g
- "DRIED SKIMMED MILK POWDER", 2.65g

Following the dairy disaggregation the first two of these ingredients are both classified as semi-skimmed milk, and the third ingredient is classified as skimmed milk.[4] If a respondent consumed 100g of "Milkshake ready to drink (e.g. Frijj, yazoo)" they would therefore consume 93.3g of semi-skimmed milk and 2.65g of skimmed milk. The 20% reduction in all dairy would then require a reduction of 18.66g of semi-skimmed milk and 0.53g of skimmed milk. As the nutrient composition of each semi-skimmed milk ingredient is slightly different

in the NDB (e.g. 0.44 kcal per 100g in "MILK SEMI-SKIMMED PASTEURISED SUMMER" versus 0.45 kcal per 100g in "MILK SEMI-SKIMMED PASTEURISED WINTER"), this reduction of 18.66g is split between the two semi-skimmed milk ingredients in proportion to their contribution to the total semi-skimmed milk content. In this example, each semi-skimmed milk ingredient contributes equally to the total semi-skimmed milk content. As such, the reduction in nutrient intake from the semi-skimmed milk reduction corresponds to 9.33g of "MILK SEMI-SKIMMED PASTEURISED SUMMER" and 9.33g of "MILK SEMI-SKIMMED PASTEURISED WINTER".

Some ingredients in the FSA recipe database were unavailable in the version of the 2022 version of the NDB. The nutrient content of these ingredients was then assigned to be that of a similar dairy ingredient. The details of the assumptions made in this assignment can be found in the supplementary material of [4].

Modelling the impact of meat and dairy substitutions

Gram-for-gram replacements for each disaggregated meat and dairy type were then applied. Meat was replaced with pulses and legumes, vegetables, eggs, oily fish, plant-based meat alternatives or poultry. Dairy milks were replaced with plant-based milks, dairy yoghurts with plant-based yoghurts and dairy solid fats with plant-based solid fats. We only considered plant-based dairy alternatives as replacements for dairy foods to be consistent with the Climate Change Committee's (CCC) 7th Carbon Budget which considered these foods to be more realistic replacements for dairy at a population level.[7] The nutritional composition of each replacement was calculated as a weighted average of the nutritional composition of foods in each food group, with each weight corresponding to the frequency of consumption of the food item in SHeS 2021.

An overview of the criteria for the inclusion of different food items in each replacement food group is provided in **Table S2**.

In the case of the yoghurt replacement, there were only two plant-based yoghurt items consumed in SHeS 2021: "Soya yoghurt, with fruit (e.g. Alpro Soya)" and "Soya yoghurt, plain (e.g. Alpro Soya)". As we only remove the nutrients from plain non-composite dairy yoghurt ingredients in the simulation, we only use the single item "Soya yoghurt, plain (e.g. Alpro Soya)" as the plant-based yoghurt substitute. For example, we only remove the nutrients contained from the non-composite dairy ingredient "Natural yoghurt, greek- style" from the SHeS item "Greek style yoghurt, fruit/honey" when applying the dairy reductions.

In the case of pulses and legumes, of all reported composite and non-composite bean items only 8% were non-composite. We therefore included the nutrient contribution of other ingredients included in composite items that contained pulses and legumes as they are much more frequently consumed in composite dishes. We then excluded all composite pulse and legume items that also contained meat, fish or dairy along with soya-derived products such as soya milk. As such, only 39% of the pulse and legume composite replacement consisted of the NDB beans disaggregated food group, due to the presence of other ingredients such as sugar in baked beans.

As there was no disaggregated food group in the NDB for plant-based meat alternatives we included all items in the sub food group “Meat alternatives incl ready meals & homemade dishes”, excluding items that also contained dairy.

Of all reported non-composite items and composite items with no dairy that contained oily fish, 75% were non-composite. Given the higher proportion of non-composite items compared to pulses and legumes, we restricted the oily fish replacement to non-composite items in the NDB oily fish food group.

Of all reported non-composite and composite items that include the NDB food group Poultry excluding dairy and other meat food groups, 16% were non-composite. Despite this lower proportion, we restricted to non-composite poultry items in the replacement as this scenario can be considered as a like-for-like substitution of meat groups.

To ensure that the food items which we included in the weighted composites were sufficiently consumed to be considered realistic meat and dairy replacements at a population level, we excluded items that were consumed by less than 1% of consumers of each replacement food group. To illustrate how the nutrient content of the weighted composites was calculated, in **Table S3** we provide the nutrient content of the foods included in the plant-based milk replacements along with the nutrient content of the weighted composite plant-based milk implemented in each simulated pathway with a dairy replacement. The foods contributing to the weighted composite replacement in each substitute food group and their associated weights are provided in **Figures S2-S9** and in the **Supplementary data**.

Table S2. Summary of the selection criteria for food and drink items included in each of the meat and dairy replacements.

Replacement	Strategy for inclusion in replacement
Meat	
Pulses and legumes	All items with non-zero disaggregated Beans, excluding all items that also contain meat, fish, dairy and those items that belong to the Recipe Sub Food Groups “Savoury Sauces Pickles Gravies & Condiments”, “Other Milk”, “Cream (Including Imitation Cream)”, “Ice Cream”, “Meat Alternatives Incl Ready Meals & Homemade Dish”, “Other Cheese” And “High Fibre Breakfast Cereals”. Manual exclusion of the item “Bolognese sauce, made with vegetarian mince (e.g. Quorn)” which contained soya-derived mince and is included in the plant-based meat alternative substitution.
Vegetables	All non-composite items in the disaggregated NDB food groups Brassicaceae, Yellow Red Green, Tomatoes and Other Veg.

Plant-based meat alternatives	All items in the recipe sub-food group “Meat alternatives incl ready meals & homemade dishes” excluding all dairy containing items.
Egg	String search for “egg” in the food description, with items that may be considered non-composite based on the item description being selected. All items that also contained dairy were then excluded.
Oily fish	All non-composite items in the NDB food group Oily Fish, excluding items that also contain dairy.
Poultry	All non-composite items in the NDB food group Poultry. No items in the list included dairy.
Dairy	
Plant-based milks	Based on manual search of plant-based milk items available in SHeS.
Plant-based yoghurt	Single item replacement: “Soya yoghurt, plain (e.g. Alpro Soya)”
Plant-based solid fats	Based on manual search of plant-based solid fat items available in SHeS

Table S3. Nutrient content per 100g of the plant-based milks included in the plant-based milk composite replacement along with the associated weighting for the weighted average for four select nutrients. All data are taken from the UK Nutrient Databank.

	Iodine (µg)	Protein (g)	Calcium (mg)	Free sugars (g)	Weight (% consumption)
**SHeS food item**					
Oat milk	0	0.94	52.33	1.69	48.8
Almond milk/ hazelnut milk	0	0.50	120.0	0.10	20.6
Soya milk, unsweetened	11.00	2.70	13.0	0.50	14.3
Soya milk, sweetened	11.00	3.00	120.0	2.80	10.5
Coconut milk, fresh (e.g. Alpro)	0	0.20	120.0	2.1	2.7
Soya milk, light	11.00	2.0	120.0	0.10	2.1

Rice milk	0	0.80	3.0	0	1.0
**Composite plant-based milk replacement**	2.96	1.31	70.44	1.27	-

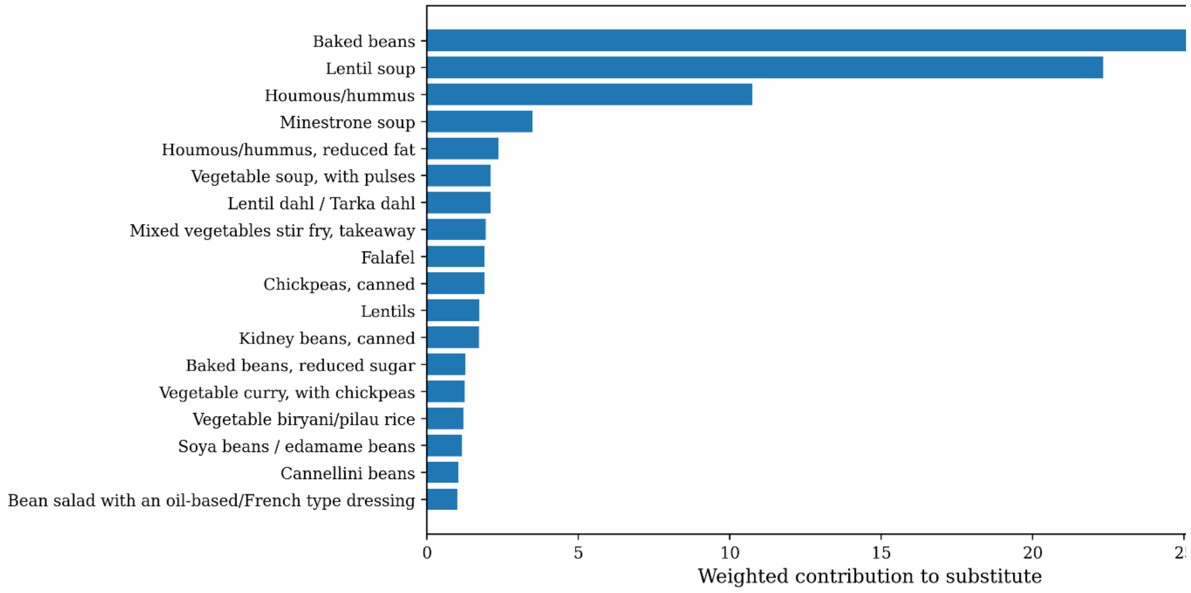

Figure S 2. Pulse and legume containing items included in the weighted composite pulse and legume replacement.

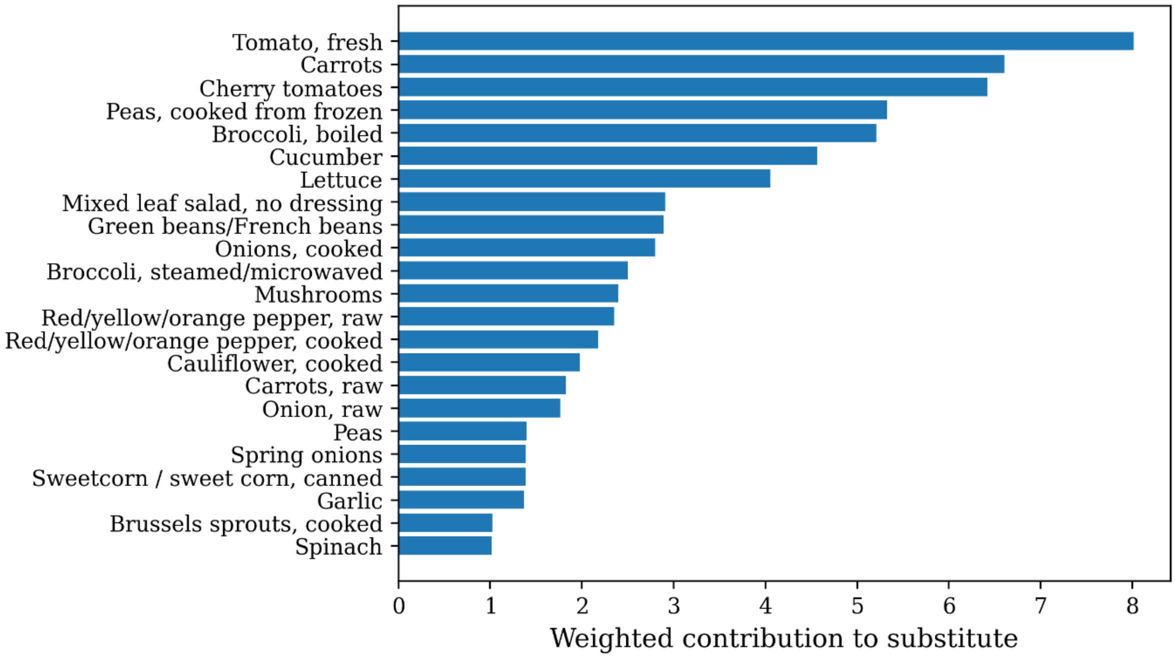

Figure S 3. Vegetable items included in the weighted composite vegetable replacement.

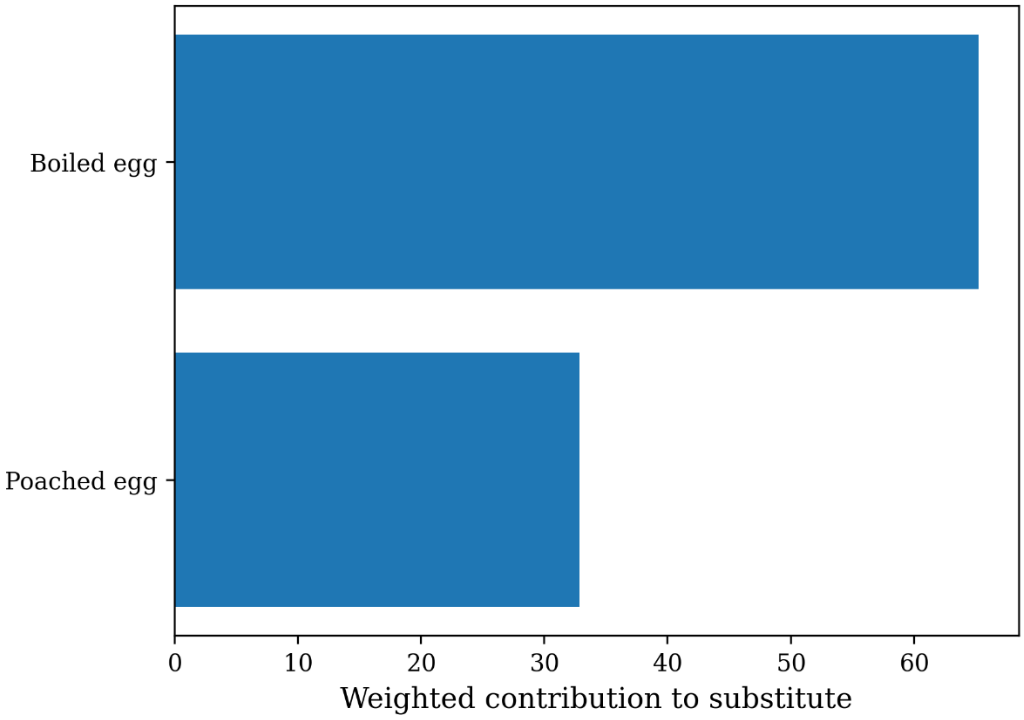

Figure S 4. Egg items included in the weighted egg composite replacement.

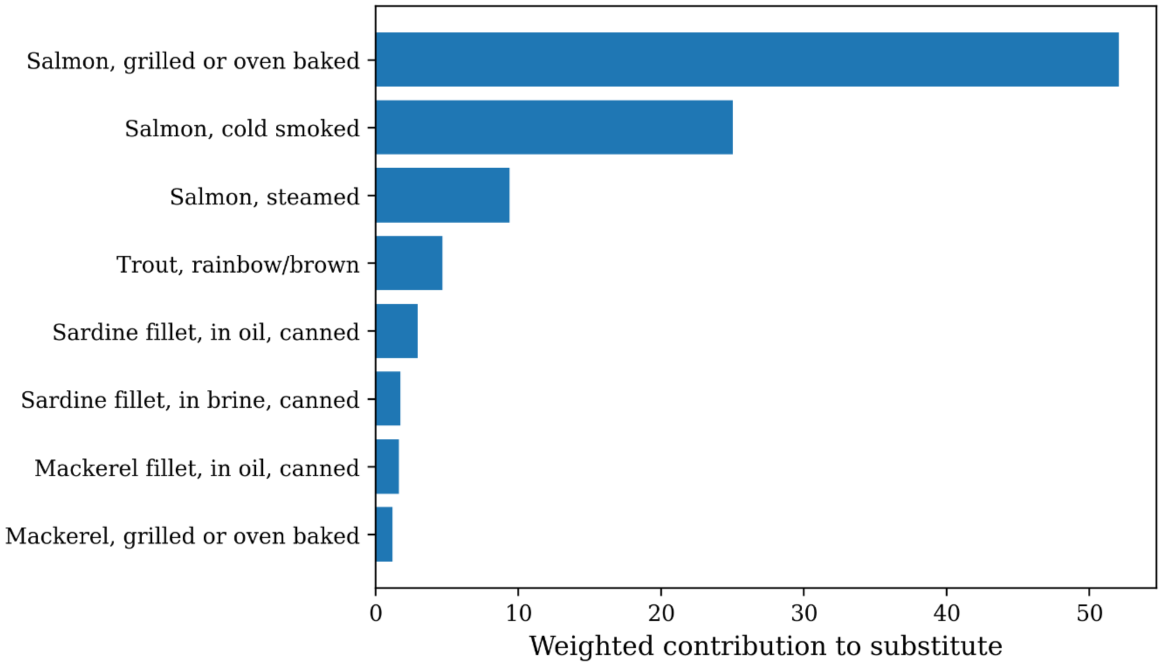

Figure S 5. Oily fish items included in the weighted composite oily fish replacement.

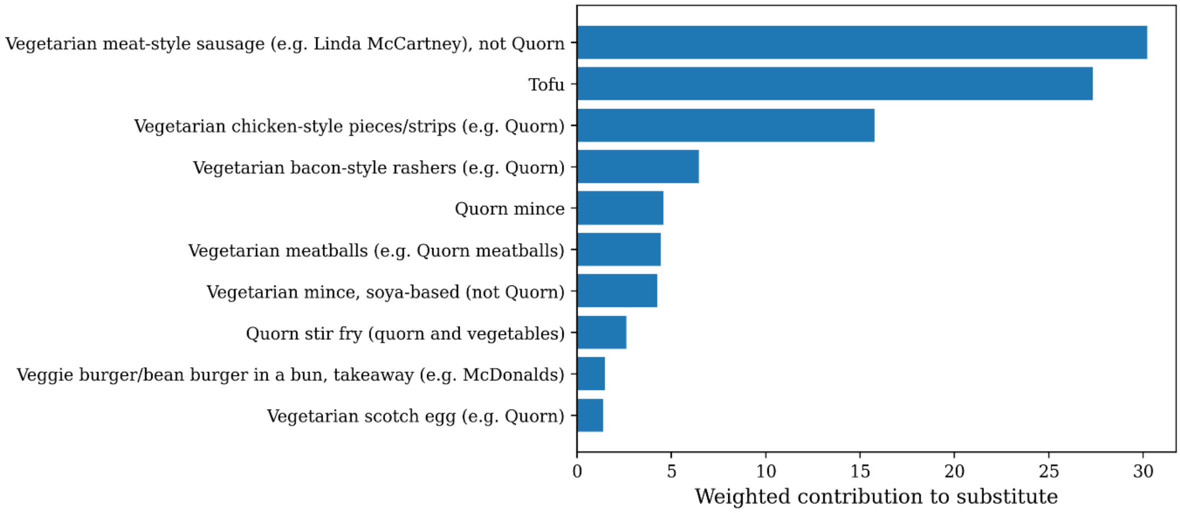

Figure S 6. Plant-based meat alternative food items included in the weighted composite of the plant-based meat replacement.

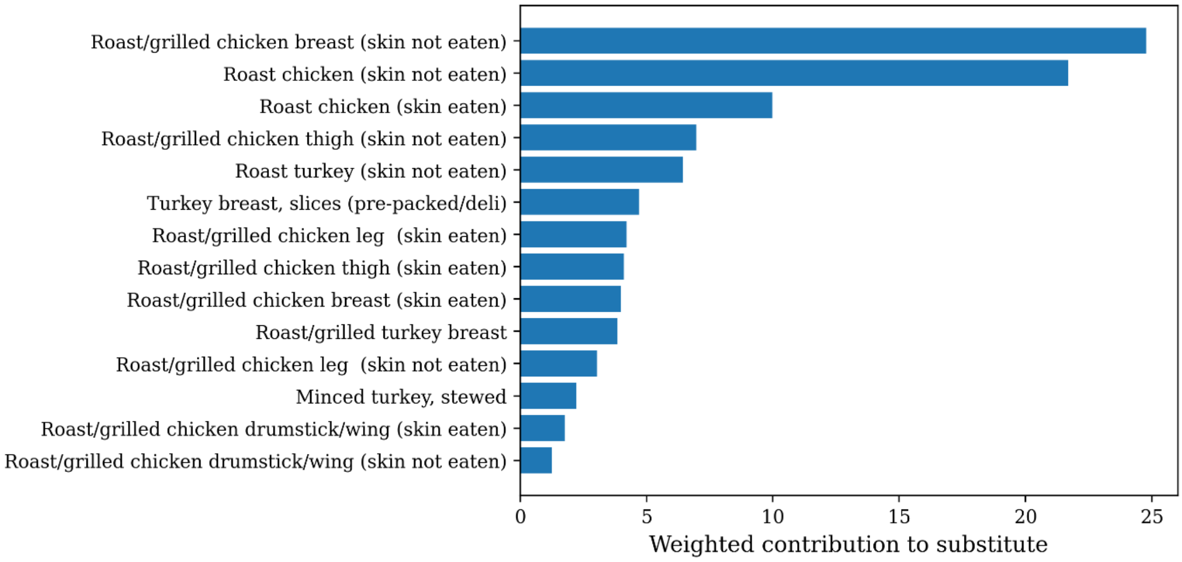

Figure S 7. Poultry food items included in the weighted composite of the poultry replacement.

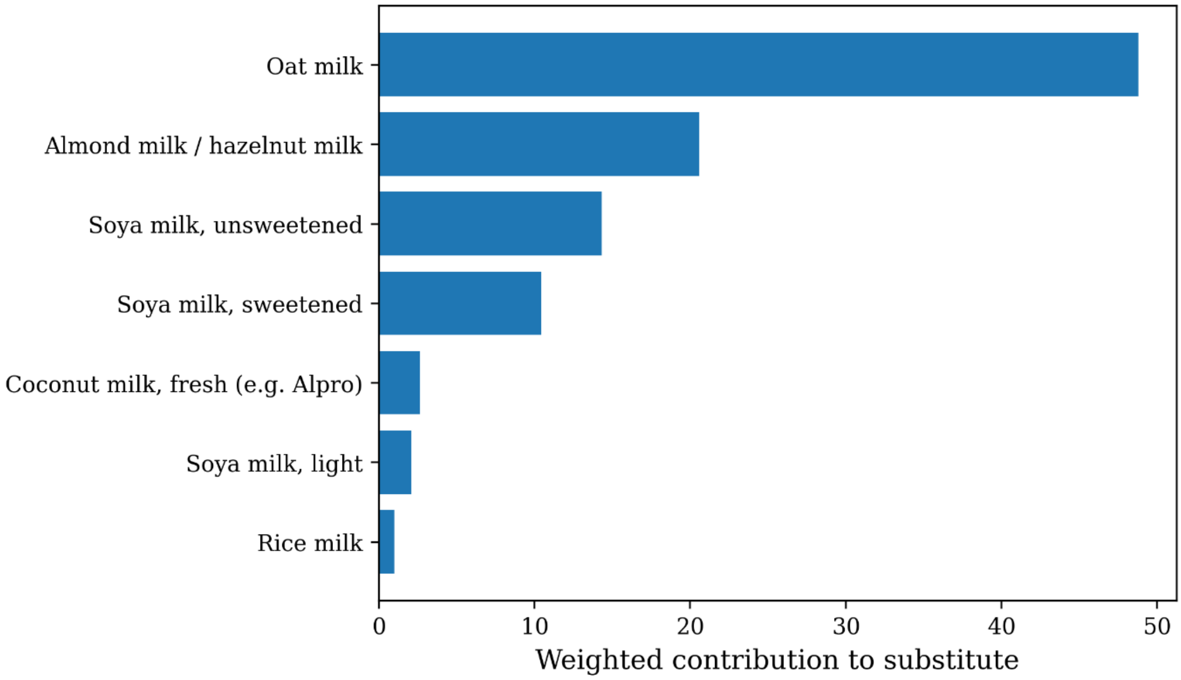

Figure S 8. Plant-based milks included in the weighted composite plant-based milk replacement.

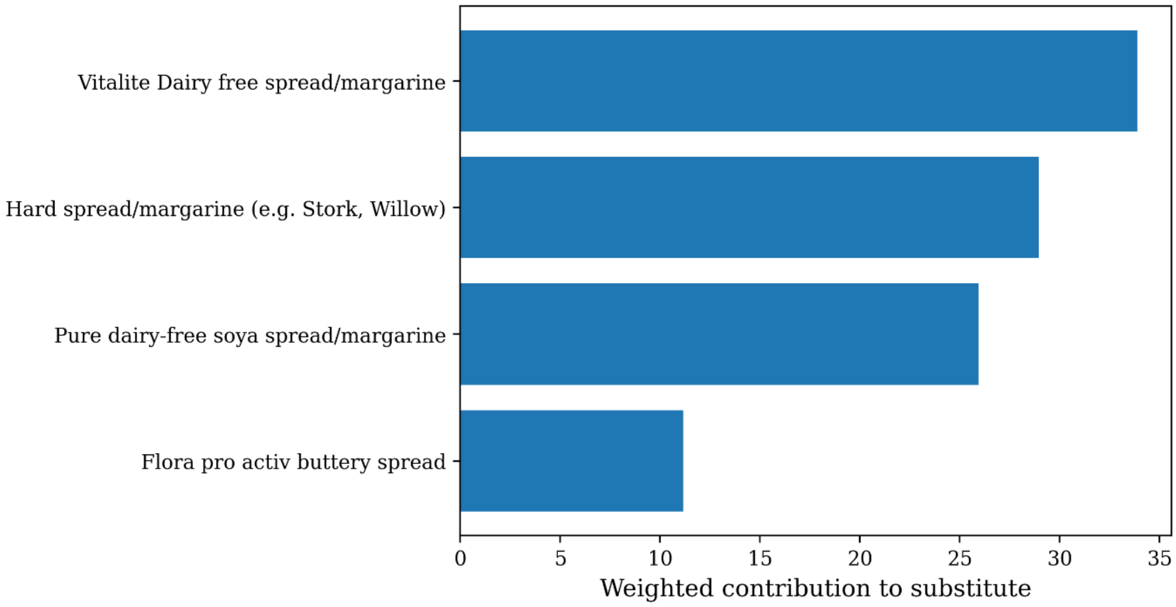

Figure S 9. Plant based solid fats included in the weighted composite plant-based solid fat replacement.

Modelling the impact on environmental outcomes and cost of diets

To estimate the impacts on environmental outcomes we matched all items in the NDB (n=2,930) to appropriate matches in foodDB, a large dataset containing the environmental impact per 100g of ~70,000 food and drink items available for purchase in major UK supermarkets [8,9], including greenhouse gas emissions, land use, water use and eutrophication. This mapping was one to many, for example, a single item "Oat milk" in the NDB was matched to all oat milks available in foodDB. Each item in foodDB is associated with a distribution of impacts for each indicator to account for the distribution of environmental impacts in the production of a single food commodity [10]. The median of this impact distribution for each indicator was taken for each matched foodDB item rather than the mean to avoid oversensitivity to outliers. The subsequent impact of the NDB item was then calculated as the average of all impacts of the matched foodDB items.

The environmental impact per gram of each NDB item was multiplied by the gram weight of the consumed item to obtain the total impact of the consumption of that item. As with nutrient intake, the total impact at the individual level was then calculated by summing over all items in the self-reported dietary data and dividing by the individual's number of recall days. In instances where the item description in the NDB and SHeS did not match, the associated food code was used to provide impact estimates for the SHeS item. If the food code was associated with multiple SHeS items, then the impact was calculated as the average of the impact of each SHeS item.

Conversion factors were also applied between the gram weight of certain drink items to account for the fact that the impacts per gram in foodDB do not include additional water from dilution. For example, the impact per gram of tea in foodDB is the impact of the tea leaves, while the impact in SHeS includes the water associated with the consumed tea. These conversion factors were derived from similar items available in The Composition of Foods Integrated Dataset (CoFID) 2021[11] available from Public Health England. To ensure the baseline per capita impact results were robust with respect to the choice of conversion factor we performed a sensitivity analysis by varying the conversion factor of each item by 20%, finding that the resultant per capita impact estimates varied by less than one percent. The conversion factors used in the analysis to convert between the foodDB impact and the associated consumption impacts in SHeS are provided in **Table S4** along with the matched item and associated derivation from CoFID.

Table S4. Conversion factors applied to convert the environmental impacts per gram of items in foodDB to the corresponding impacts per gram of the reported items in SHeS 2021

SHeS 2021 items	Conversion factor	Derivation from matched items in CoFID
• Tea<br>• Herbal/ Fruit tea<br>• Decaf tea<br>• Green Tea	0.015	"Tea, black, infusion, average", assuming 15g leaves per litre water
• Coffee, fresh<br>• Decaf coffee, fresh	0.0515	Average of "Coffee fresh weak infusion" (34g

		ground coffee per litre) and "Coffee fresh strong infusion" (69g ground coffee per litre), resulting in 51.5g of ground coffee per litre.
• Coffee, instant<br>• Decaf coffee, instant	0.089	"Coffee, instant, made up with water", Calculated from 2g instant coffee to 225ml of water.
• Hot / drinking chocolate, made with water, low calorie	0.09	"Drinking chocolate powder, made up with semi-skimmed milk", assuming the same ratio of chocolate powder to water as chocolate powder to milk, with 18g powder to 200ml of milk.
• Blackcurrant squash / juice, no added sugar, diluted<br>• Ribena squash / juice, diluted, with ADDED SUGAR<br>• Ribena squash / juice , diluted, no added sugar<br>• Blackcurrant squash , high juice, no added sugar, diluted	0.125	Based on "Blackcurrant juice drink/squash, diluted", derived from assuming 38g concentrate to 250ml of water.
• Orange squash, no added sugar, diluted<br>• Fruit squash / juice, no added sugar, diluted<br>• Orange squash / juice, with ADDED SUGAR, diluted<br>• Orange squash, high juice, no added sugar, diluted<br>• Fruit squash / juice, with ADDED SUGAR, diluted<br>• Apple and blackcurrant squash,	0.25	Based on conversion factors of "Fruit juice drink/squash, diluted", "Fruit juice drink/squash, no sugar added, diluted" and "Lime juice cordial, diluted" which all assume 50g concentrate to 200ml of water.

no added sugar, diluted

- Fruit squash, high juice, no added sugar, diluted
- Cranberry squash, diluted
- Orange squash, hi juice, diluted
- Fruit squash, high juice, with ADDED SUGAR, diluted
- Red berry squash, diluted
- Elderflower cordial, with ADDED SUGAR, diluted
- Lime cordial, no added sugar, diluted
- Lime cordial, diluted

As foodDB also contains price information it was possible to estimate total daily expenditure in SHeS along with the associated impact on expenditure in each simulation pathway. In foodDB price data were taken from 2022, 2021 and 2019. Price data from 2022 were chosen, if available. For items that did not have 2022 price data, 2021 price data were assigned (n=53), while if 2021 price data were not available then 2019 price data were assigned (n=6). In addition, any items that were priced greater than £5 per 100g were excluded from the NDB to foodDB mapping in estimating the price of NDB items (n=1,238). This cutoff was chosen as it was the 96th percentile in the distribution of prices in foodDB and ensured that any items that overestimated the price due to parsing errors or mislabelling were excluded from the analysis.

Environmental impact adjusted for usual dietary intake

Self-reported dietary data is typically underreported [12] and, as a result, all estimates for per capita impacts, cost and nutrient intake should be considered conservative. To obtain a more reliable estimate for the environmental impact of each indicator at the population level we combined the number of underreported calories for each individual based on recommended daily energy intake for their age and sex with the environmental impact per calorie of that individual. This approach assumes that the underreported food and drinks are similar in composition to the self-reported items. For example, if an individual had a total daily dietary GHG of 5kgCO_2e day based on 1500 kcal, their emissions per calorie would be 0.003 kgCO_2e/kcal. Age and sex dependent recommended daily calorie intakes were based on UK dietary reference values[13]. In this example, if the individual is male, then their adjusted daily emissions estimate is given by 5 + (0.003 × 1000) = 8.33 kgCO_2e. The total yearly estimate is then computed by taking the individual's adjusted daily impact, multiplying by the sample weight, summing over the whole population and multiplying by 365. As the sample weight provided in SHeS is a relative sample weight which averages to one across the sample, before the summation each sample weight was multiplied by a scale factor derived from Scottish Census Data [14] to ensure that the sum of the sample weights was equal to the Scottish adult (16+) population size in 2021. The calorie adjusted total yearly impact for each indicator is given in **Table S5.**

Table S5. Estimated total environmental impacts over a year for the Scottish adult population, adjusted for underreported calorie intake.

**Indicator**	**Yearly impact, adjusted for daily caloric intake (95% Uncertainty Interval)**
GHG, MtCO_2e	10.4 (10.3, 10.6)
Land use, $10^3 \times km^2$	13.8 (13.6, 14.0)
Water use, $10^{11} \times$ litres	10.6 (10.4, 10.7)
Eutrophication, $10^9 \times$ gPO_4e	40.6 (40.1, 41.2)

Uncertainty estimation

The uncertainty intervals in the per capita outcomes and changes in outcomes in all simulation pathways were computed by combining several sources of uncertainty (uncertainty estimation for health outcomes is described at the end of the "Modelling the impact on chronic diseases, mortality and obesity" section below). For all nutrients, standard errors were calculated using the R "survey" package [15] to account for the complex survey design, with the upper and lower uncertainty range estimated as +/- 1.96 multiplied by the standard error. In the case of environmental outcome and expenditure, we further accounted for the within-item uncertainty for each NDB item from the standard deviation of the median impact values of all matched foodDB items.

In instances where a SHeS food code corresponded to multiple items in the NDB, the within-item impact uncertainty for that item was calculated as follows. If there are M items in the NDB corresponding to a single food code in SHeS, the combined error on the impact per gram of indicator I is estimated by

$$\sigma_I = \frac{1}{M} \cdot \sqrt{\sum_{j=1}^{M} \sigma_{Ij}^{\;\;2}}$$

where σ_{Ij} is the standard error in the impact per gram of item j. The final estimate for the baseline standard error of the indicator associated with the consumption of each item in the dietary data was calculated by multiplying the standard error per gram by the gram weight of consumption. Standard errors were combined at each simulation stage as follows.

For all non-composite meat and dairy items and meat and dairy containing items with a non-zero reduction of a disaggregated meat or dairy type, the standard error of the item's impact after removing meat and dairy was computed as $\sqrt{\sigma_{item}^2 + \sigma_{ingredient}^2}$ where σ_{item} is the standard error of item's impact prior to applying the reductions (e.g. the standard error of impact of "Beef lasagne") and $\sigma_{ingredient}$ is the standard error of the impact of the meat or dairy ingredient that being deducted from the original item (e.g. "Minced beef, stewed"). The value of $\sigma_{ingredient}$ was calculated by taking the standard error of the impact per gram of the ingredient, multiplied by the gram weight of the reduction. For non-composite items, σ_{item} and $\sigma_{ingredient}$ are related by $\sigma_{ingredient} = \alpha \times \sigma_{item}$, where α is the ratio between the gram weight of the reduction and the original gram weight of the item. For example, if the original consumption level of a non-composite meat or dairy item was 100g and the reduction was 20g in a particular scenario, then $\alpha = 0.2$.

The standard error of the impact of all items consumed per individual in SHeS was then calculated as

$$\sigma_{Individual} = \frac{1}{N} \cdot \sqrt{\sum_{j=1}^{M} \sigma_j^2}$$

where N is the number of days of recall, M is the total number of food and drink items consumed across both days of recall and σ_j is the standard error of the indicator of item j.

In the three pathways where the CCC targets are met through reduction in red meat alone, there was additional uncertainty associated with the variable impact of achieving the reduction via reductions in different red meat food groups. As the process was repeated for 50 iterations there were 50 different values for $\sigma_{Individual}$ for different realisations of red meat reduction combinations per individual. The final estimate for the impact uncertainty for each individual prior to substitution was calculated as

$$\sigma_{Individual,pre\ sub} = \frac{1}{50}\sqrt{\sum_{j=1}^{50} \sigma^2_{Individual,j}}$$

where $\sigma_{Individual,j}$ is the standard error of the individual's total impact for Monte Carlo iteration j.

As each meat and dairy replacement other than poultry and plant-yoghurts consisted of a weighted composite of different food items within that food group, with weights associated with consumption frequency in SHeS, the standard error of the replacement's impact also must account for these weights. The final standard error per gram of the substitute was calculated as

$$\sigma_{Sub\ per\ gram} = \frac{1}{\sum_{i=1}^{N} w_i}\sqrt{\sum_{i=1}^{N}(w_i \times \sigma_i)^2}$$

where w_i is the weight of the food item *i* in the weighted average of N total items included in the composite replacement, each with a standard error of the impact per gram σ_i. In all substitution pathways the standard error on the environmental impact in each of the substitution pathways is computed by combining the individual standard error post reduction with the standard error of the meat or dairy replacement as follows

$$\sigma_{Individual,post\ sub} = \sqrt{\sigma^2_{Individual,pre\ sub} + \sigma^2_{Sub}}$$

where σ_{Sub} is $\sigma_{Sub\ per\ gram}$ multiplied by the gram weight of the total meat or dairy reduction that the substitute replaces. For example, should an individual experience a 20g reduction in red meat across all red meat food types and replace the red meat with eggs, then σ_{Sub} for a given indicator would be 20 multiplied by $\sigma_{Sub\ per\ gram}$ of the corresponding indicator. Each individual now has a standard error for each indicator associated with the simulated diet.

The standard per capita error of each indicator of the baseline diet were computed in the same manner as $\sigma_{Individual}$, but without any of the steps involving meat and dairy reductions and substitutions. The standard within-item error of the per capita impact for each indicator X can then be calculated as

$$\sigma_X = \frac{1}{\sum_{i=1}^{N} sw_i} \sqrt{\sum_{i=1}^{N} \left(sw_i \times \sigma_{i,X}\right)^2}$$

where N is the number of individuals in SHeS, sw_i is the survey sample weight of individual i and $\sigma_{i,X}$ is the standard error of indicator X for individual i. The within item standard error σ_X for indicator X can then be combined with the standard error on the per capita impact associated with the complex survey design $\sigma_{X,survey}$ to obtain the standard error of per capita impacts as $\sqrt{\sigma_X^2 + \sigma_{X,survey}^2}$.

The outcome after following each of these steps for all simulation pathways is the standard error on the per capita impacts for each indicator. The standard error on the difference of each outcome between the baseline and each simulated pathway was computed as the square root of the sum of the squares of the standard error in the baseline and the simulated pathways. The uncertainty intervals in the **Supplementary data** were computed as +/- 1.96 multiplied by the standard error of the difference.

Food category contributions to baseline impacts, cost and nutrient intake

After estimating the nutrient content, environmental impact and cost of all food and drink items in SHeS 2021, it was possible to estimate the relative contribution of each major food group to nutrient intake, environmental impact and cost at baseline. In this case food group refers to the high-level food categories provided in SHeS (e.g. “Meat and meat products”), rather than the disaggregated meat and dairy types employed in the simulation (e.g. “Beef”). Therefore, the contribution of each food group to each indicator includes that of all ingredients in that food group. For example, the contribution to dietary GHGs from the SHeS food group “Non-alcoholic beverages” includes both hot drinks such as lattes and all sweetened and sugary beverages.

To estimate the contribution of each food group to each indicator we first calculated the relative contribution of all items in each food group to the total value of the indicator for each participant, before averaging over the number of recall days. For example, say on day one an individual consumed 2mg of zinc from 10g of the food group “Milk and milk products” while on day two the individual consumed 3mg of zinc from the food group “Meat and meat products” and this was their only source of zinc across both days. Averaged over both days, the proportion of zinc intake from “Milk and milk products” is therefore 1mg/2.5mg = 0.4, and the proportion of zinc intake from “Meat and meat products” is 1.5mg/2.5mg = 0.6. This approach was preferred over taking the average of the ratios of the proportional contribution of each food group to an indicator across each day of recall as it is more reflective of long-term intake [16]. Two approaches were then taken to calculate the relative proportion of each food group to nutrient intake compared to environmental indicators and cost. In the case of nutrient intake, we took the sample weighted average of the proportional contribution of each food group to nutrient intake across all participants. For environmental impacts and cost, we took the proportion at a population level by summing the average daily impact across all individuals from a single food group before dividing by the total population level impact across all food groups. While the difference between the two approaches is usually minor [17], the distinction is warranted by the fact that population level outcomes are of more interest for environmental indicators while per capita outcomes are of more interest for nutrient intake. Given that the relative contribution of different food groups to nutrient intake is an average of proportions rather than itself being a proportion, in instances where a nutrient is consumed episodically such as with haem iron or beta-cryptoxanthin, the sum of the proportions across all food groups does not necessarily equal one. However, in most instances the proportions can be interpreted as a percentage contribution to total intake. We therefore have multiplied the proportional contributions of each food group by 100. We provide the relative contribution of all main food groups to nutrient intake, environmental impact and cost in the **Supplementary data**.

Modelling the impact on chronic diseases, mortality and obesity

To derive estimates for the impact on type 2 diabetes (herein diabetes), cardiovascular disease (CVD), all-cause mortality and obesity, we adapted a microsimulation model previously

developed for the US, micro Simulation of the Health Impact of Food Transformation, (mSHIFT) [18] to the SHeS 2021 data. While a comprehensive discussion of the methodology used in calculating disease incidence is provided in the supplementary material of [18], in this section we provide an overview of the model as well as details on how the model was adapted for this study.

A combination of demographic and health data in SHeS were used as input to previously developed risk models for diabetes and CVD to estimate each individual's baseline disease risk [19,20]. This baseline risk was then adjusted to account for each respondent's unprocessed red meat, processed red meat and total dairy intake by multiplying the baseline risk by a relative risk associated with the intake of each respective food group. Unprocessed red meat and processed red meat intake were all assigned a dose-response relative risk association for diabetes and CVD risk, taken from a meta-analysis and an analysis of six large US-based cohort studies [21,22], with higher intakes of both food groups being associated with a higher risk of developing each disease. Total dairy intake was assigned a relative risk association with diabetes only [23], with higher total dairy consumption being associated with a reduced risk of diabetes. Sampling distributions were assigned based on the published relative risk associations for different levels of intake to estimate the mean and confidence interval at each intake level. In this study, the sampling distributions used to calculate the relative risk of each disease associated with unprocessed red and processed red meat were the same as those implemented in [18]. The relative risk of diabetes associated with total dairy intake was further estimated by fitting a beta distribution to the relative risk for different total dairy intake levels.

Mortality risk were estimated based on age and sex specific yearly mortality estimates for 2019 from National Records Scotland [24].

mSHIFT requires that the sample weights used in the model are scaled such that the sum over all the sample weights is equal to the population size rather than the sample size. The sample weights used in this analysis were the weights assigned to individuals with self-reported dietary intake data "SHeS_Intake24_wt_sc", which were provided in SHeS as relative sample weights such that their sum is equal to the sample size (n=3,447). Census data from the National Records of Scotland [14] were used to calculate the total adult population size (16y+) in 2021 resulting in 4,563,378 individuals. The scale factor needed to transform the relative sample weights to absolute sample weights can then be calculated by taking the ratio of the total population size to the sample size resulting in 1325.32. The relative sample weights in SHeS were then all multiplied by 1325.32 to obtain an estimate of their absolute size. All respondents who reported being pregnant (n=16) were removed from the analysis.

A summary of all variables required by mSHIFT to estimate is provided in **Table S6**, including the health outcome for which each variable is required, the prevalence of missing data for each variable and, in the case of missing data, the approach taken to impute the missing data. As SHeS 2021 does not contain data on parental history of diabetes, we used the variable indicating if the respondent has a parent, sibling or child with diabetes in its place.

Table S6. Summary of the SHeS variables used in mSHIFT to predict health outcomes.

Simulation Module	Individual Characteristic	Variable Specification	SHeS Variable(s)	Proportion of Simulated Population Missing Values	Imputation strategy
Diabetes, CVD, Mortality	Age	Continuous years	age	0%	N/A
Diabetes, CVD, mortality, weight	Sex	Categorical:1/0, female/ male	Sex	0%	N/A
Diabetes	Ethnicity	Categorical: 1/0. Five ethnicity variables, with 1 assigned to one and only one of 'White: Scottish', 'White: Other British', 'Asian', 'White: Other', 'Other minority ethnic' and 0 otherwise for each ethnicity variable.	Ethnic05	0.9%	No ethnicity assigned to those who did not report an ethnicity
Diabetes, CVD	Smoking status	Categorical: 1/0, smoker/non-smoker	Cignow	0%,	N/A

CVD	Type-2 diabetes status	Categorical: 1/0, diabetes/no diabetes	Type2	0.3%	Respondents who reported "Don't know" were assigned a value of 0
Diabetes, weight	Weight	Continuous kg	SlfWtDV_adj	8.1%	Bayesian symbolic regression*
Diabetes, weight	Height	Continuous cm	SlfHtDV_adj	4.6%	Bayesian symbolic regression*
CVD	Taking blood pressure medication	Categorical: 1/0, taking blood pressure medication/ not taking blood pressure medication	Medcinbp	0.06%	Respondents who reported "Don't know" were assigned a value of 0
Diabetes	High systolic blood pressure	Categorical: 1/0, high blood pressure (>140mmHg)/no high blood pressure	Currbp	0.4%	Respondents who reported "Don't know" were assigned a value of 0
Diabetes	Parental history of diabetes	Categorical: 1/0, yes/no	FamDB	0.8%	Respondents who reported "Don't know" or "refused" were assigned a value of 0
Weight	Minutes of moderate physical activity per day	Continuous, minutes	hrwalk10R, actwktime	0.08%, 0.3%	Respondents who reported "Don't know" or were "Non-applicable" were assigned a value of 0

Weight	Minutes of vigorous physical activity per day	Continuous, minutes	MVPA10wk, hrshwk10, Hrsman10	0%, 0%, 0%	N/A
Diabetes, CVD	Systolic blood pressure	Continuous mmHg	N/A	100%†	Bayesian symbolic regression*
Diabetes, CVD	Total cholesterol	Continuous mg/dL	N/A	100%†	Bayesian symbolic regression*
CVD	HDL cholesterol	Continuous mg/dL	N/A	100%†	Bayesian symbolic regression*

* The full details of how Bayesian symbolic regression was used as an imputation model are provided later in this section

† These data are imputed using a model developed using data from three rounds of the US based National Health and Nutrition Examination Survey, with details provided later in this section.

Yearly incidence estimates were obtained by multiplying each respondent's disease risk by their sample weight and summing the result over all individuals. Where available, observed incidence estimates for different demographic groups were used to calibrate the final risk estimate to ensure that predicted incidence matches observed incidence in the first simulation year in that demographic group, while ensuring that the relative incidence between individuals with different risk profiles within that group remained consistent with their individual risk profiles. Data diabetes incidence in 2021 from the 2021 Scottish Diabetes Survey[25] were used to calibrate diabetes risk in 10-year age bands. As an approximation we assumed that the diabetes incidence in the youngest age group 16-19 was equal to that of the age group 10-19 and was the average of the 2018 and 2020 values. CVD incidence data were taken from open access Public Health Scotland heart disease and stroke incidence data for 2021-2022.[26] In **Table S7** and **Table S8** we provide the number of diabetes and CVD cases in each demographic group recorded from the national statistics and that predicted in the first year of the simulation.

Table S7. Estimated number of new diabetes cases in 2021 from [25], along with that predicted by mSHIFT after dividing the final risk the calibration constant in the final column, averaged over 50 computations of the disease incidence.

Age Group (years)	Estimated diabetes incidence	Average predicted diabetes incidence after one year of the simulation, and the associated 95% uncertainty interval	Calibration constant
**16-19**	20	19, (13, 26)	82.51
**20-29**	293	293, (243, 342)	17.46
**30-39**	1254	1253, (1097, 1409)	5.73
**40-49**	3010	3014, (2668, 3361)	1.74
**50-59**	5976	5988, (5228, 6749)	1.78
**60-69**	5884	5862, (5134, 6591)	1.75
**70+**	5773	5761, (5086, 6436)	1.17

Table S8. Estimated number of new CVD cases per year from Public Health Scotland data [26], along with that predicted by mSHIFT after dividing the final risk the calibration constant in the final column, averaged over 50 computations of the disease incidence.

Age Group (years)	Estimated CVD incidence	Average predicted CVD incidence after one year of the simulation, post calibration and the associated 95% uncertainty interval	Calibration constant
Males			
**0-44**	593	592, (545, 639)	3.62
**45-64**	5582	5574, (5119, 6029)	1.79
**65-74**	4349	4345, (3960, 4730)	1.61
**75+**	5709	5716, (5125, 6306)	1.01
Females			
**0-44**	260	259, (238, 280)	5.21
**45-64**	2519	2530, (2331, 2729)	2.11
**65-74**	2610	2611, (2392, 2831)	1.40
**75+**	6389	6415, (5607, 7223)	0.64

We further estimated the impact of the pathways with no replacement on each individual's body mass index (BMI). Following the reduction in meat and dairy intake each individual experiences a decrease in the average daily caloric intake. This change in caloric intake was then used as an input to an established model that estimates the change in body weight for a given change in caloric intake, taking into account age, sex, height, weight and physical activity levels[27]. The specific implementation was the linearized model for long-term weight change associated with a drop in daily caloric intake using equation (11) in the supplementary material of Hall et al.[27]As the change in diet was assumed to occur in the first year, the age used in the body weight model was the participant's age at baseline. Physical activity was divided into four daily categories: daily minutes of vigorous physical activity, moderate physical activity, sedentary non sleep/light physical activity and sedentary sleep. The SHeS variables that were assigned to each physical activity category are provided in **Table S6**. As self-reported physical activity levels are typically overreported[28,29] we performed several pre-processing steps on the physical activity data. First, we combined moderate and vigorous activity into a single variable and capped it at a total daily value at 180 minutes in accordance with guidance from the International Physical Activity Questionnaire[30]. We then applied the calibration equations of Welk et al.[29] to reduce the over-reporting bias in the self-reported

values based on age, sex and BMI. Specifically, we applied the alternative calibration model in Welk et al. that did not include education level as a covariate as the study population was US based. As self-reported vigorous physical activity is more reliable than self-reported moderate physical activity[31], but does not typically exceed 30 minutes per day at population level[32,33] we then randomly assigned a new value for daily minutes of vigorous physical activity between 0 and 30 before re-allocating the difference from the total recalibrated value to moderate physical activity. Daily minutes of sedentary non-sleep were calculated as the difference between 1440 minutes per day, the combined daily minutes of moderate and vigorous physical activity and 7 hours of sleep. Metabolic equivalent scores associated with each physical activity category were sampled from shifted beta distributions provided in **Table S6,** with ranges taken from the MET scores associated with physical activities in each category[34]. Beta distributions were chosen to favour activities with lower MET scores in each category, which we assumed to be a better measure of daily activity over a ten-year period. Each sampled MET score was then adjusted for each participant using the correction factors derived by Kozey et al.[35] based on baseline energy expenditure and initial body weight to account for the fact that the same activity may be associated with different MET scores for individuals with different resting metabolic rates. The final physical activity level was then calculated using the methods provided in Gerrior et al.[36] by combining estimates for baseline energy expenditure and the adjusted MET scores. This individual level physical activity level was then combined with the estimate of each participant's resting metabolic rate which was derived using the equations from Mifflin et al.[37] to calculate the physical activity parameter δ which is defined in the supplementary material of Hall et al.[27] and included in the body weight model. For each iteration of the simulation, we assumed that the daily minutes of physical activity remained constant over time. If a respondent did not include data on a particular moderate or vigorous physical activity, then these data were assumed to be zero (n=12 respondents).

Table S9. Sampling distributions for the metabolic equivalent (MET) scores associated with different physical activity categories, with the beta distribution *Beta*(α, β) defined by the shape parameters α and β.

Physical activity	Sampling distribution
Vigorous physical activity	$6.0 + Beta(2,\ 5)$
Moderate physical activity	$3.0 + Beta(2, 5)$
Sedentary, not sleep	$1.1 + 1.5 \times Beta(2.5, 6)$

Three biomarker variables required for both the diabetes and CVD risk prediction models were not available in SHeS: systolic blood pressure, total cholesterol and high-density lipoprotein (HDL) cholesterol which were not collected in SHeS, while height and weight data were missing for <10% of individuals (**Table S6).** Following a review of various imputation strategies we used Bayesian Symbolic Regression (BSR) to develop a model to impute these variables based on demographic and health data that was not missing[38]. After assigning a target variable and a set of predictive variables, BSR performs a stochastic search among viable analytic closed-form mathematical expressions of the predictive variables that describe their relationship to the target variable. The cost function associated with each model is given by the description length, which penalises overly complex models. The output

provides a series of closed form mathematical models obtained from a stochastic search of the viable model space, with model with the minimum description length after 10,000 Monte Carlo samples used to impute the missing data.

BSR has been shown to outperform other regression approaches such as Gaussian Processes, as well as performing well on noisy and sparse datasets [38]. It is also able to capture potential nonlinear relationships between the source and target variables. However, a key motivation for using BSR was the interpretability of the imputation model. Furthermore, as the imputation model is an analytical function, imputed values for missing variables can be updated in the simulation throughout each simulation year in a way that is both interpretable and quantifiable. This method has been applied to various domains such as carbon capture technology [39] and the prediction of country-level energy use based on socioeconomic data [40], with the full details of the algorithm as well as the source code available from Guimerà et al[38].

To develop the biomarker imputation models, it was necessary to obtain a dataset that contained both the missing biomarker data and variables that were also available in SHeS. To this end, we combined data from the 2013, 2015 and 2017 rounds of the US based National Health and Nutrition Examination Survey (NHANES) (n=18,362) to be used as training data for the BSR algorithm. After excluding individuals that did not have data for all training variables the final dataset included 14,291 unique individuals. To test the performance of the imputation models for systolic blood pressure, HDL cholesterol and total cholesterol on an out-of-sample distribution we took data from the 2021 round of NHANES. After removing all individuals with missing data for the variables used in the imputation models, the final out-of-sample test set consisted of 5,496 unique individuals.

As a smaller number of individuals were missing height data (n=157) and weight data (n=280) in SHeS, data from other SHeS participants with height and weight data were used as input to the BSR algorithm for these two variables. Due to the fact that, among the 157 participants with missing height data, there were 151 participants that were also missing weight data, weight was not included as a variable in the height imputation model and vice versa. As ethnicity was included as a predictive variable in the imputation model for both height and weight, we did not use US data as an out-of-sample test set, but rather data from the UK National Diet and Nutrition Survey (NDNS) years 12-15. Two variables in NDNS did not completely overlap with SHeS 2021, namely ethnicity and whether the participants reported having high blood pressure. As the predictive variables related to ethnicity in the height and weight imputation models derived from SHeS 2021 were "White Scottish" and "White Other British", in the out-of-sample test set we assigned these both to be based on the single binary NDNS variable "ETHGRP2" which records the participant as either "White" or "Non-White" ethnicity. As there was no variable that recorded whether the participant in NDNS has high blood pressure, we used the variable "BPMEDD2" which recorded whether the participant was taking medication for high blood pressure as a proxy variable for high blood pressure in the out-of-sample test set.

As the output of the algorithm fits a fixed value of each model parameter, for a given set of input variables the predictions are completely deterministic. To provide uncertainty estimates in the imputation model predictions arising from the uncertainty in the imputation model parameters, we applied a Markov Chain Monte Carlo (MCMC) algorithm with a NUTS sampler[41] on the model with the minimum description length implemented with python's PyMC library (version 5.28.1)[42] to derive a joint posterior distribution for the model

parameters. For each model parameter we assigned weakly informative priors consisting of truncated normal distributions to ensure the sign remained consistent and set the standard deviation to be 20% of the maximum likelihood value from the initial point estimates from BSR. The likelihood was set to be gaussian with the prior on the observational uncertainty assigned to be a half-normal distribution with a standard deviation set to that of the standard deviation of the corresponding variable in the training data (e.g. σ =20mmHg for systolic blood pressure). Two independent chains were run for each imputation model with 1000 samples for the burn-in and a subsequent 1000 sampling iterations. For a given iteration of the simulation, the samples from the imputation model's posterior distribution were fixed throughout the ten-year period with the parameter posteriors re-sampled for each subsequent iteration. The uncertainty interval associated with each health outcome therefore accounts for the additional uncertainty associated with the missing biomarker data by marginalising over plausible values of each missing variable for each individual. In **Table S10** we provide a summary of the notation used in each imputation model. Although SHeS 2021 does not contain systolic blood pressure data, there were data on whether the individual had high blood pressure, defined as a systolic blood pressure greater than 140mmHg. This was used to define the new variable X_{HBP} in the NHANES training dataset based on the systolic blood pressure data in NHANES. We did not include income, education or ethnicity data in the BSR algorithm due to potential demographic differences between the US and Scotland.

Table S10 Overview of the variables used as input to the Bayesian Symbolic Regression (BSR) algorithm and those that BSR included in the final imputation models.

Variable	Notation	Data type, unit
Variables available in both SHeS and NHANES		
High blood pressure diagnosis	X_{HBP}	Binary (1: Doctor diagnosed high blood pressure, 0: otherwise)
Taking medication for high blood pressure	X_{MBP}	Binary (0: No; 1: Yes)
Age	X_A	Integer, years
Sex	X_S	Binary (0: Male, 1: Female)
Body mass index (BMI)	X_{BMI}	Continuous, $\frac{kg}{m^2}$
Weight*	X_W	Continuous, kg
Height*	X_H	Continuous, cm
Smoker	X_{Sm}	Binary (0: Non-smoker, 1: Smoker)
Diabetes	X_D	Binary (0: No diabetes, 1: diabetes)
"White, Other British" ethnicity**	X_{WOB}	Binary (0: No; 1: Yes)
"White, Scottish" ethnicity**	X_{WS}	Binary (0: No; 1: Yes)
Imputed variables (available in NHANES and not in SHeS)		

Systolic blood pressure (SBP)	X_{SBP}	Continuous, mmHg
High-density lipoprotein cholesterol (HDL)	X_{HDL}	Continuous, mg/dL
Total cholesterol (TC)	X_{TC}	Continuous, mg/dL

*Missing data on height and weight were imputed by using the available height and weight data in SHeS to train the BSR algorithm.
** We only label ethnicities that appeared in the final imputation model. All ethnicities were included in the height and weight imputation model search.

The systolic blood pressure model with the minimum description length was a five-parameter model with five independent variables, given by

$$X_{SBP} = Exp\left(\beta_4 \left(\frac{X_W}{\beta_1 + \beta_2 X_A} + \beta_0 + \sqrt{X_A} \right) + \beta_3 X_{HBP} \right),$$

and with maximum likelihood parameter values given by $\beta_0 = 67.93$, $\beta_1 = -0.93$, $\beta_2 = 1.88$, $\beta_3 = 0.059$ and $\beta_4 = 0.063$. A comparison between the predictions of this model and the training data in NHANES is provided in **Figure S10**. The R^2 of the model was 0.30 on the sample data, while the R^2 on the out-of-sample data was 0.17. The model captures the increase in estimated systolic blood pressure associated with a high blood pressure diagnosis through the parameter β_3 along with an increase in SBP with age and body weight.

(A)

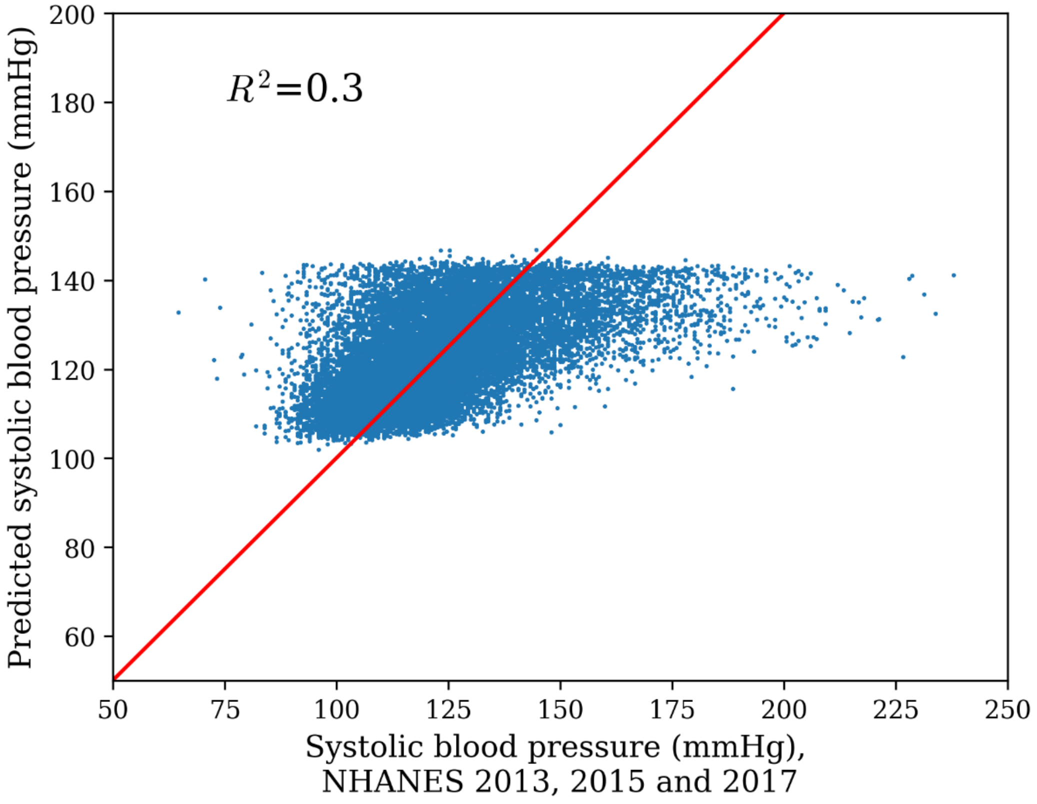

(B)

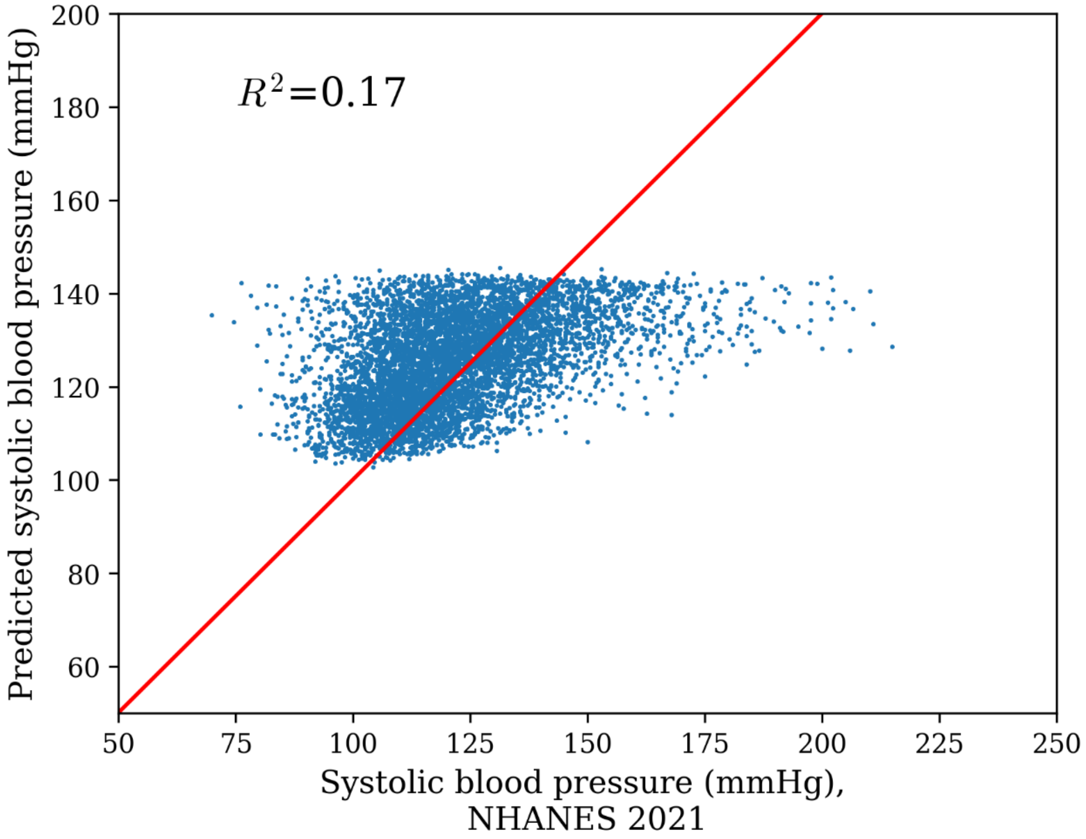

Figure S 10. Comparison between the predicted values for systolic blood pressure and (A) the measured values from the NHANES 2013, 2015 and 2017 (n=17,269) used in the BSR algorithm and (B) NHANES 2021 (n=6,391) which was used as an out-of-sample test set.

In **Figure S11** we show the distribution of predicted systolic blood pressure sampled from the posterior distribution of the model parameters for three individuals in SHeS 2021, one aged 32 and two aged 64 with and without a high systolic blood pressure diagnosis. There is significant variability in the predictions for each participant, however the distributions shift in accordance with what would be expected from the model, increasing with both age and high blood pressure. The fact that the shape of each distribution is similar indicates that the variability in the model prediction is dominated by the observational error in the likelihood rather than variation in the model parameters.

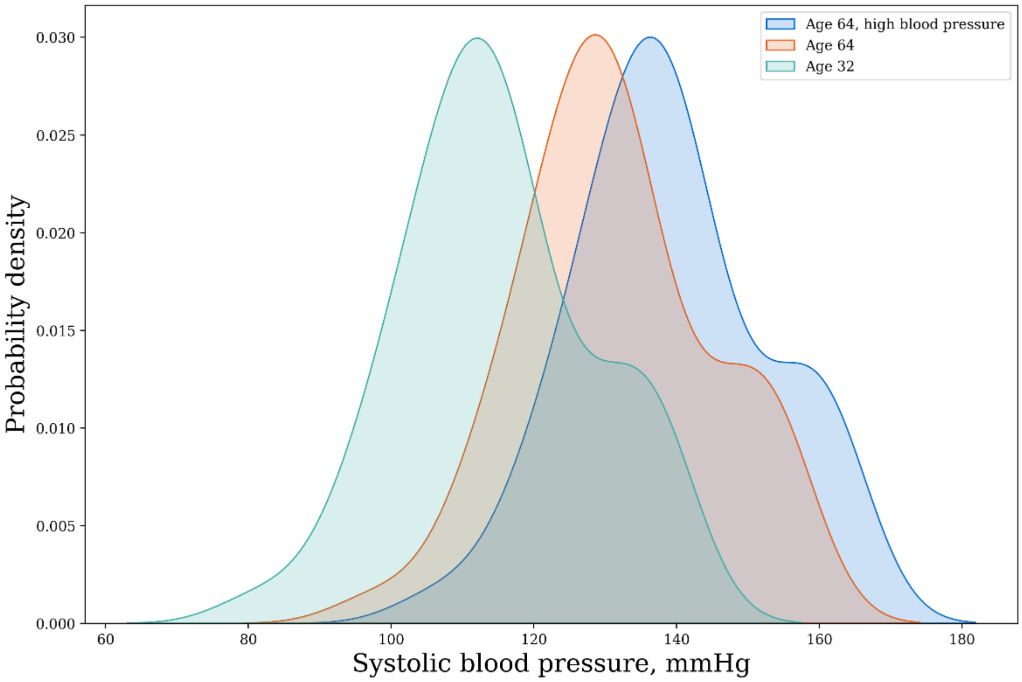

Figure S 11. Distribution of predicted values for systolic blood pressure for three individuals in SHeS after taking samples from the parameter posterior distribution of the systolic blood pressure imputation model. SBP: Systolic blood pressure

To demonstrate how the model predictions vary for different participants in each simulation year, **Figure S12** shows the variation in systolic blood pressure with age for different fixed values of the other independent variables in the imputation model: high blood pressure diagnosis and weight with the model parameters are set to their maximum likelihood values. In all instances, systolic blood pressure increases with at a similar rate beyond the age of 30 but is systematically shifted to larger values for increases in body weight as well as doctor diagnosed high blood pressure.

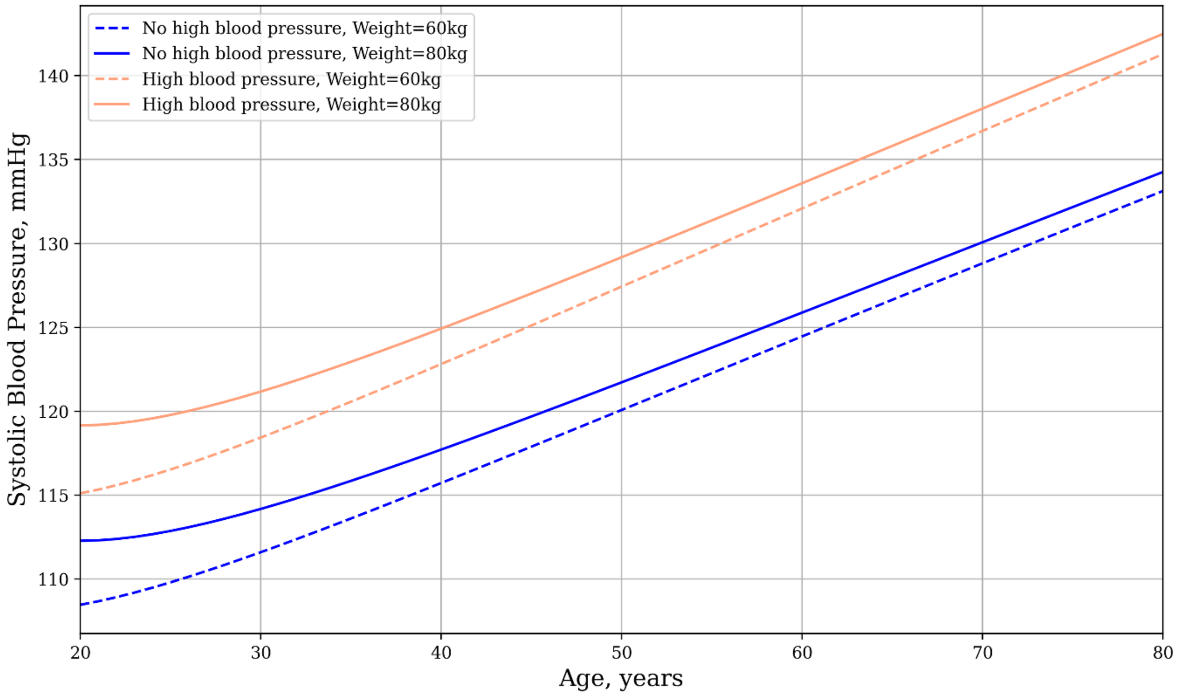

Figure S 12. Variation of systolic blood pressure with age according to the systolic blood pressure imputation model for six individuals in SHeS. The imputation model parameters are set to their maximum likelihood values. SBP: Systolic blood pressure

The final prediction model with the minimum description length for HDL cholesterol was given by the following six-parameter model with five variables

$$X_{HDL} = \left[\beta_1 \cdot \left(\beta_2^{X_A} + \beta_3^{\tilde{X}-1}\right)\right]^{\beta_4^{X_D}} - \beta_0 - X_{Sm} ,$$

where

$$\tilde{X} = (\beta_1 \cdot X_S)^{Exp[\beta_5 \cdot X_{BMI}]} .$$

The corresponding values for each parameter are given by $\beta_0 = 74.5$, $\beta_1 = 8 \times 10^{-15}$, $\beta_2 = 0.98$, $\beta_3 = 0.48$, $\beta_4 = -0.15$ and $\beta_5 = -0.15$. The performance of this model was inferior to the systolic blood pressure prediction model with an R^2 of 0.21 on the training data and an R^2 of 0.24 on the out-of-sample data, indicating that the health and demographic data used to impute HDL cholesterol are less informative than those of systolic blood pressure (**Figure S13**).

(A)

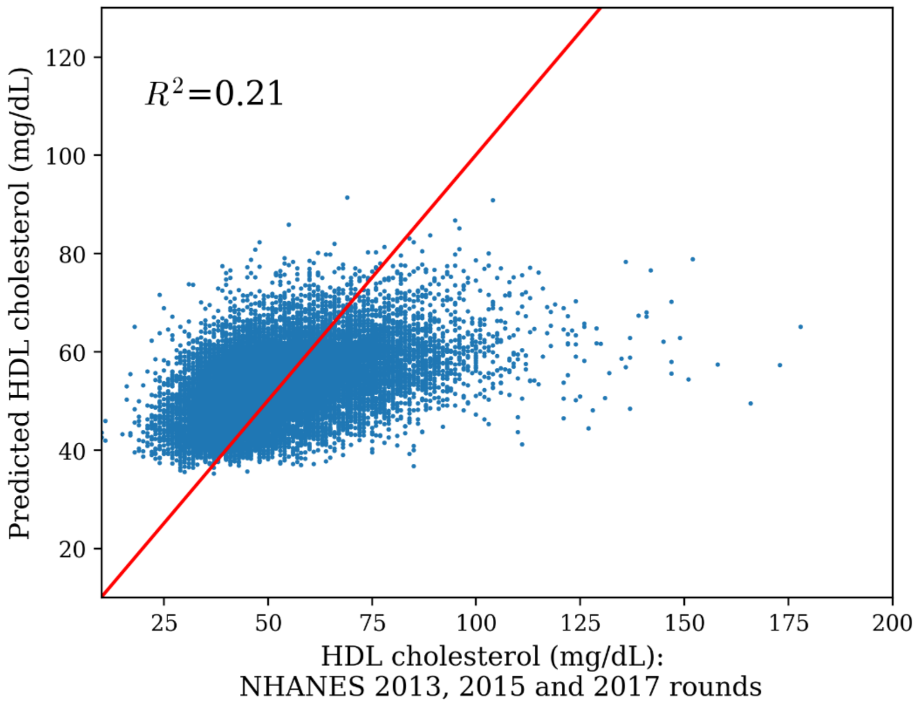

(B)

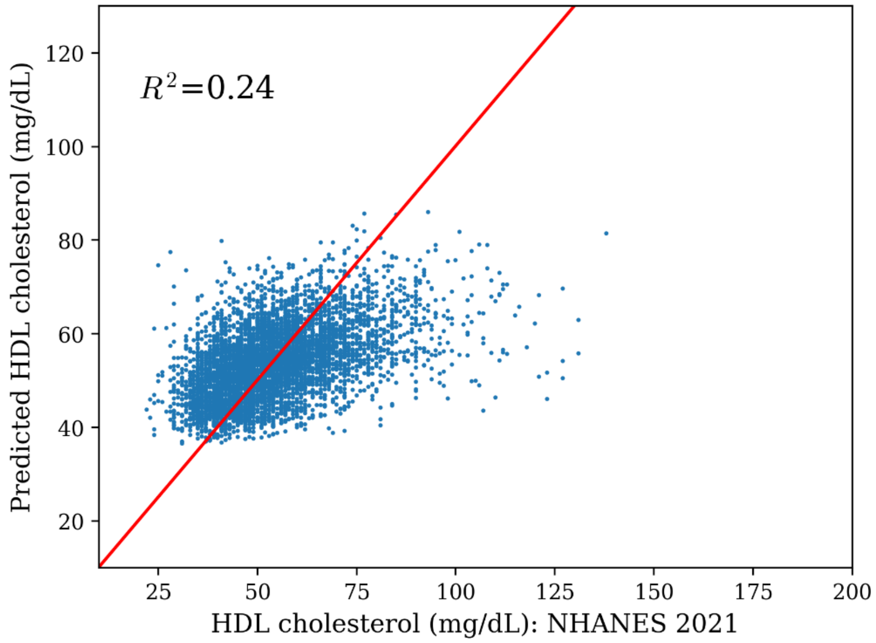

Figure S 13. Predicted levels of HDL cholesterol from the imputation model against (A) the measured values from the NHANES 2013, 2015 and 2017 (n=18,362) used in the BSR algorithm and (B) NHANES 2021 (n=5,720) which was used as an out-of-sample test set.

In **Figure S14** we show how the model varies with age for six different demographic groups. On average, as age increased so did HDL cholesterol levels. Females also have higher HDL cholesterol levels regardless of differences in smoking status and BMI, with a female with BMI=30kg/m^2 having a higher level of HDL cholesterol than a male with BMI=25kg/m^2 across all ages. Smokers of the same sex have lower HDL cholesterol levels in the model relative to non-smokers.

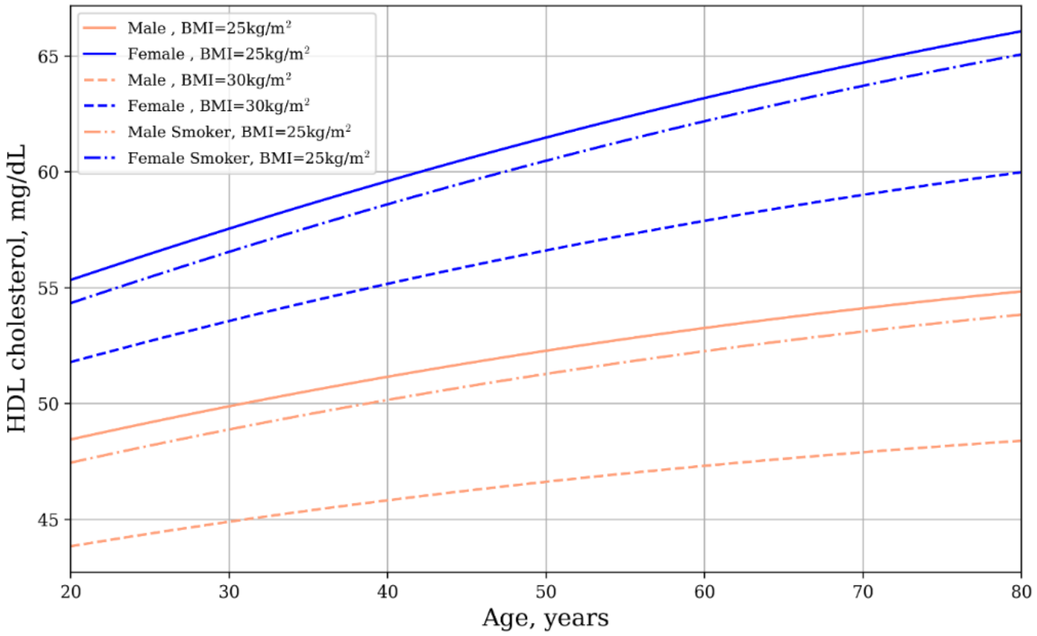

Figure S 14. Variation of HDL cholesterol levels with age according to the HDL imputation model for six participants in SHeS, demonstrating the difference by sex, BMI and for smokers vs non-smokers.

The model for total cholesterol with the minimum description length was given by the following nine-parameter model with nine variables

$$X_{TC} = \frac{\beta_0}{X_A^{\tilde{X}}} \cdot \left[\beta_1 + \beta_2 \cdot \left(\beta_3 + \beta_4^{X_S}\right) \cdot \left(X_{MBP} + X_D\right)\right]^{\tilde{X}}$$

where

$$\tilde{X} = \beta_5 + \beta_6 \cdot X_A \cdot [\beta_7 \cdot (X_{MHP} + X_D)]^{\beta_8 + \beta_9 \cdot X_{HDL}}$$

The maximum likelihood parameter values for each parameter were $\beta_0 = 4.7$, $\beta_1 = -6.4$, $\beta_2 = -4755.1$, $\beta_3 = 0.04$, $\beta_4 = -0.05\beta_1$, $\beta_5 = 0.8$, $\beta_6 = 0.04$, $\beta_7 = 0.004$, $\beta_8 = 0.1$ and $\beta_9 = -0.001$. The performance of this model on the training data is comparable to that of HDL cholesterol with a R^2 of 0.22 while the performance of the model on the out-of-sample data was inferior with an R^2 of 0.16 (**Figure S15**). HDL cholesterol biomarker data in NHANES were included in the training data for the total cholesterol model. As a result, total cholesterol also depends on the imputed HDL cholesterol model and was therefore imputed after the HDL cholesterol imputation for each individual. This approach ensured that the imputed values for total cholesterol were consistent with the imputed values of HDL

cholesterol, at the expense of including extra uncertainty in the final total cholesterol model predictions from the variability in the uncertainty in HDL cholesterol.

(A)

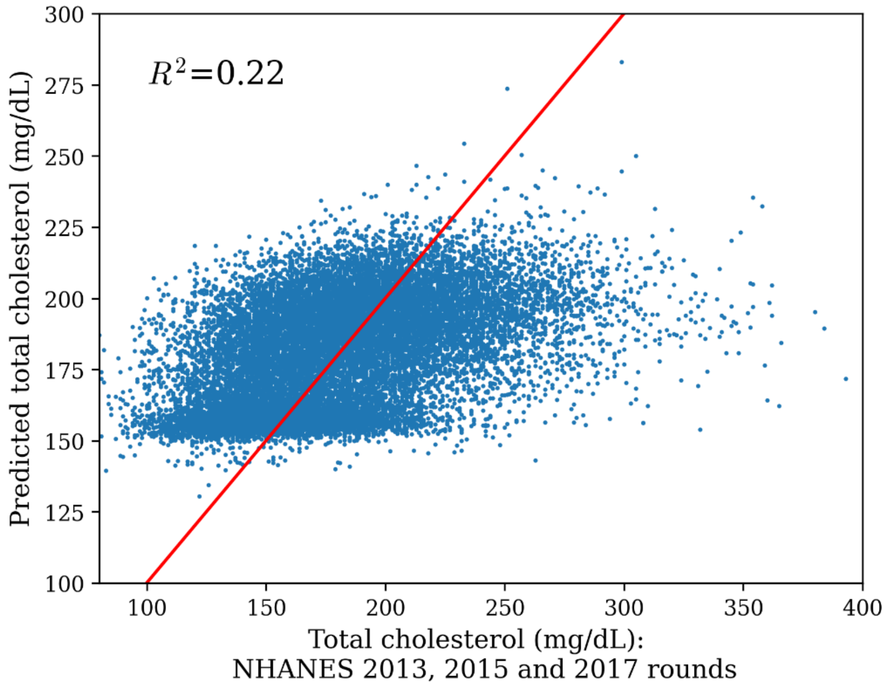

(B)

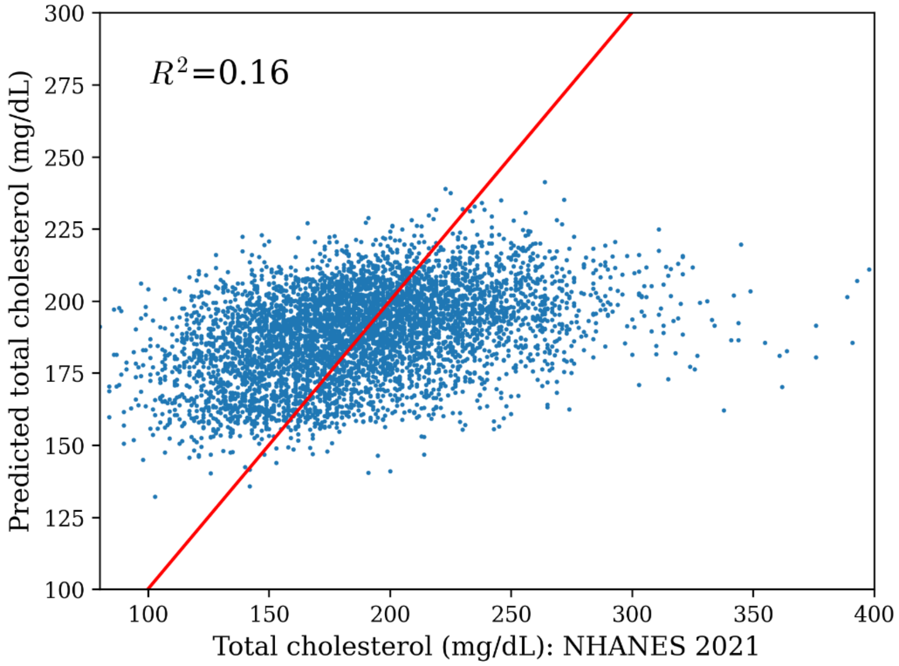

Figure S 15. Predicted levels of total cholesterol from the imputation model against (A) the measured values from the NHANES 2013, 2015 and 2017 (n=18,362) used in the BSR algorithm and (B) NHANES 2021 (n=5,720) which was used as an out-of-sample test set.

In **Figure S16** we show the variation of total cholesterol with age for the maximum likelihood estimates for the posterior parameters for six individuals. The model captures a nonlinear association between age and total cholesterol, increasing with age until approximately 55yr before subsequently decreasing with age. Females typically have higher total cholesterol levels than males. In addition, those that report taking blood pressure medication have lower total cholesterol levels than those of the same age, sex and weight that do not take blood pressure medication.

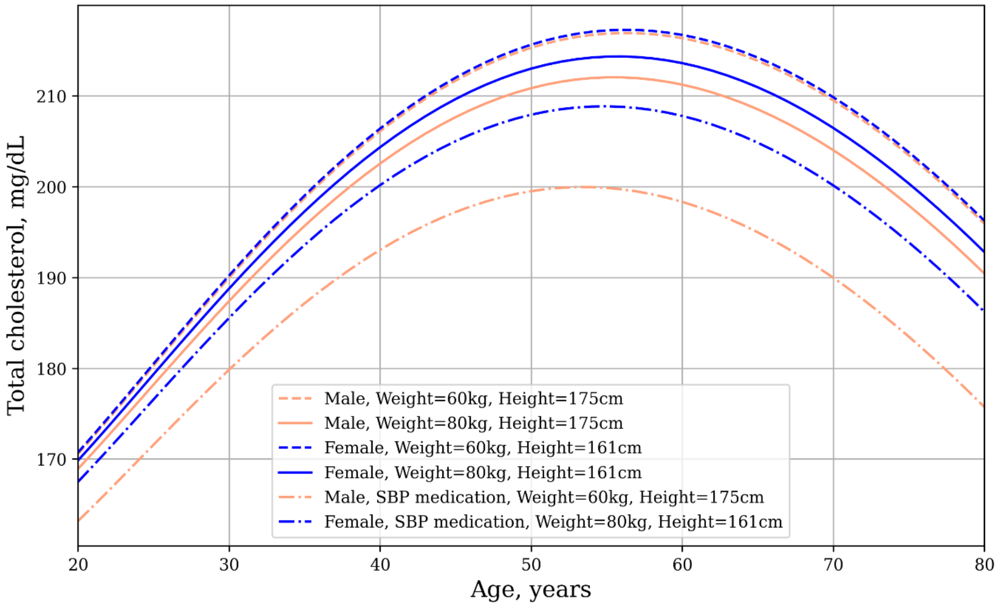

Figure S 16. Variation of total cholesterol with age for six SHeS participants, showing the difference for different ages, weights and whether the respondent reports taking blood pressure medication. SBP: Systolic blood pressure

Despite the fact that the R^2 for each imputation model is below 0.4, the uncertainty in the imputation model predictions are accounted for in the uncertainty estimates of disease risk and the associated disease incidence. To illustrate this point, in **Figure S17** we show how the distribution in imputed values for systolic blood pressure (panel A), HDL cholesterol (panel B) and total cholesterol (panel C) impacts the distribution in the estimate of yearly CVD baseline risk (panel D) for six participants from SHeS 2021 across the 50 iterations of the simulation. To aid comparability, all participants were chosen to be female, non-diabetic and have a BMI less than 30 kg/m^2. The low predictive power of the systolic blood pressure, HDL and total cholesterol models results in significant overlap in the predictive distributions. However, this does not necessarily translate into significant overlap in CVD risk due to the dependence on other demographic variables in the risk model. As can be seen in **Figure S17 Panel D**, the width of the distribution in yearly CVD baseline risk for these participants varies by age, with younger participants having both lower average risk and variation in risk than older participants. The risk distribution in **Figure S17 Panel D** solely reflects the uncertainty in the imputation models and does not account for the additional uncertainty associated with the unprocessed red and red processed meat intake of each participant.

(A)

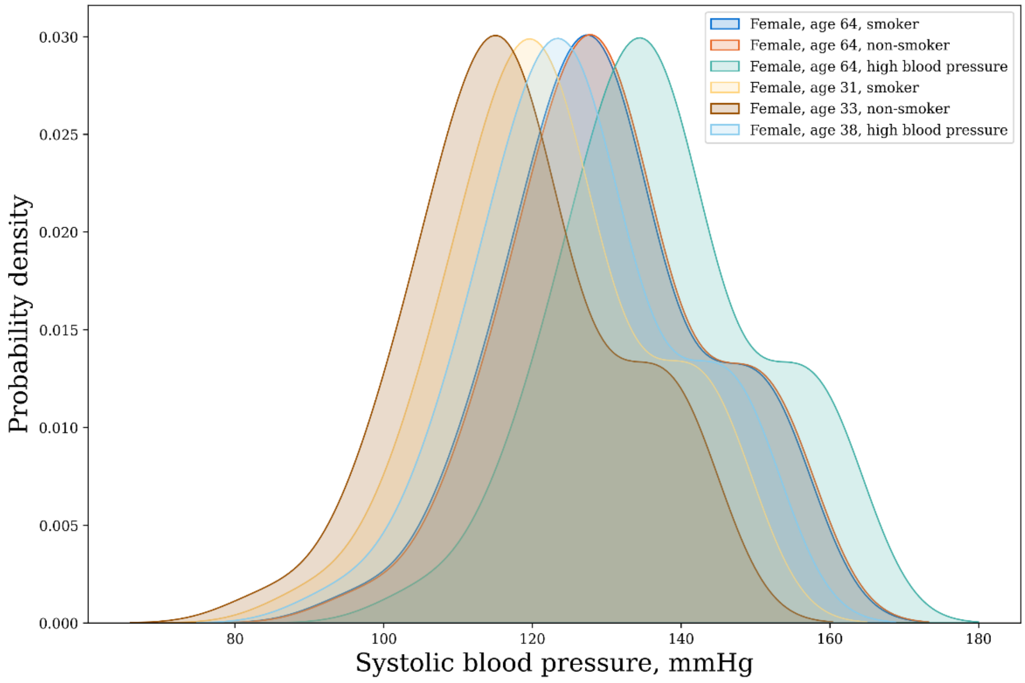
Female, age 64, smoker
Female, age 64, non-smoker
Female, age 64, high blood pressure
Female, age 31, smoker
Female, age 33, non-smoker
Female, age 38, high blood pressure
Probability density
0.000
0.005
0.010
0.015
0.020
0.025
0.030
80
100
120
140
160
180
Systolic blood pressure, mmHg

(B)

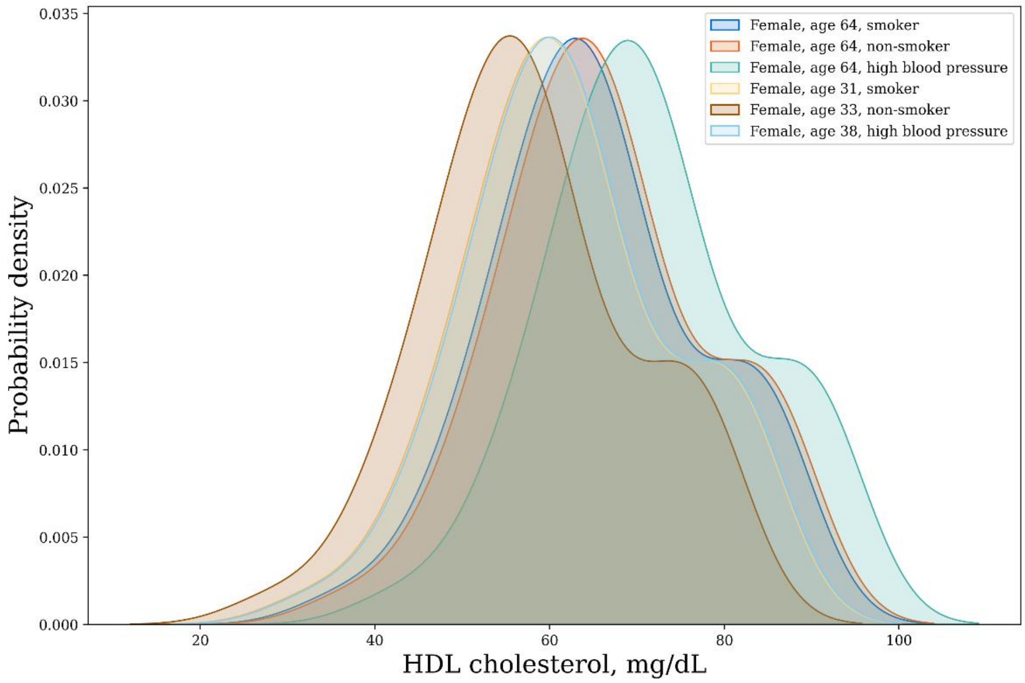
Female, age 64, smoker
Female, age 64, non-smoker
Female, age 64, high blood pressure
Female, age 31, smoker
Female, age 33, non-smoker
Female, age 38, high blood pressure
Probability density
0.000
0.005
0.010
0.015
0.020
0.025
0.030
0.035
20
40
60
80
100
HDL cholesterol, mg/dL

(C)

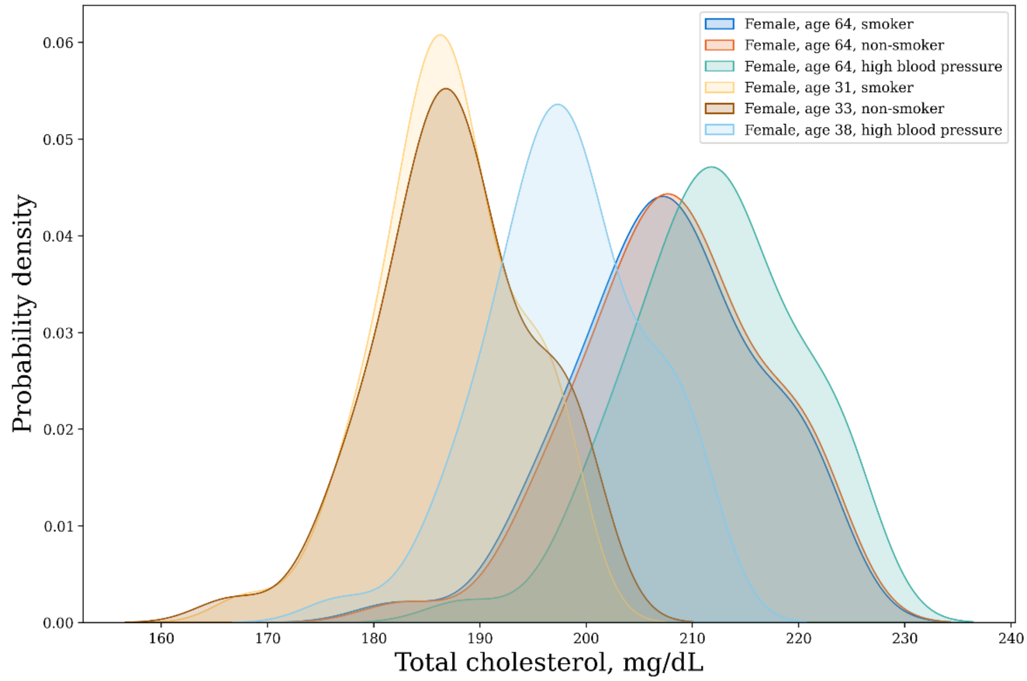

Female, age 64, smoker
Female, age 64, non-smoker
Female, age 64, high blood pressure
Female, age 31, smoker
Female, age 33, non-smoker
Female, age 38, high blood pressure
Probability density
Total cholesterol, mg/dL
0.00
0.01
0.02
0.03
0.04
0.05
0.06
160
170
180
190
200
210
220
230
240

(D)

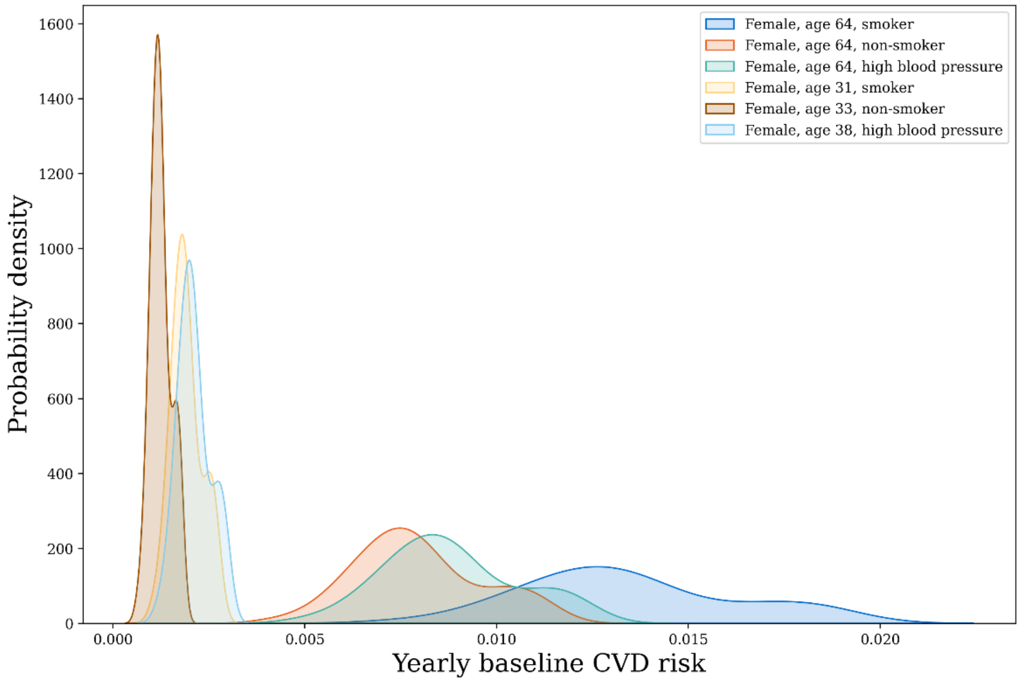

Female, age 64, smoker
Female, age 64, non-smoker
Female, age 64, high blood pressure
Female, age 31, smoker
Female, age 33, non-smoker
Female, age 38, high blood pressure
Probability density
Yearly baseline CVD risk
0
200
400
600
800
1000
1200
1400
1600
0.000
0.005
0.010
0.015
0.020

Figure S 17. Variation in systolic blood pressure (A), HDL cholesterol (B), total cholesterol (C) and the resultant estimate of baseline yearly CVD risk distributions (D) for six female, non-diabetic participants from SHeS 2021 with a BMI less than 30.

Height and weight data were missing for 157 and 280 respondents in SHeS 2021, respectively. Given that height and weight data were available for the remaining respondents in SHeS, these data were used as input to the BSR algorithm to develop an imputation model for those missing height and weight data. As these data were taken from respondents in Scotland, in this instance we included ethnicity and SIMD (Scottish Index of Multiple Deprivation) data as the input to the BSR algorithm. Missing values for height and weight were imputed at the start of the simulation and the imputation models were not used to update their values in different simulation years. The height model with the minimum description length was given by the following three-parameter model with three variables

$$X_H = \beta_0 + \beta_1 \cdot X_A^2 + X_S(\beta_2 + X_{WOB})$$

where X_{WOB} equals one if the respondent reports an ethnicity of "White, Other British" and zero otherwise. The parameter values at the minimum description length were given by $\beta_0 = 180.15$, $\beta_1 = -0.001$ and $\beta_2 = -13.96$. The negative value of β_1 indicates that height is lower in older individuals and the negative value of β_2 indicates that females have a smaller height value than males on average. This model performs reasonably well with R^2 equal to 0.58 on the training data (**Figure S18, Panel A**) and an R^2 of 0.46 on the out-of-sample data (**Figure S18, Panel B**).

The weight imputation model with the minimum description length was given by the following six-parameter model based on age, sex and whether a participant reported taking blood pressure medication

$$X_W = \beta_0 + \beta_1 X_S + \beta_2 X_A + \beta_3 X_S X_A + \beta_4 X_A^2 + \beta_5 X_{MBP}$$

The maximum likelihood parameter values are given by $\beta_0 = 64.60$, $\beta_1 = -5.95$, $\beta_2 = 0.95$, $\beta_3 = -0.13$, $\beta_4 = -0.0097$, $\beta_5 = 6.86$. The model performs poorly relative to the height imputation model with an R^2 of 0.19 on the training data (**Figure S18, Panel A**) and an R^2 of 0.16 on the out-of-sample data (**Figure S18, Panel B**).

(A)

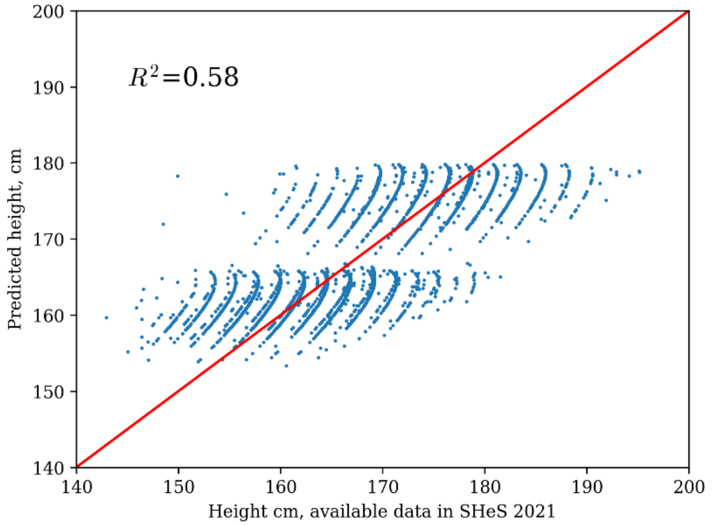
R^2=0.58
Predicted height, cm
Height cm, available data in SHeS 2021
200
190
180
170
160
150
140
140
150
160
170
180
190
200

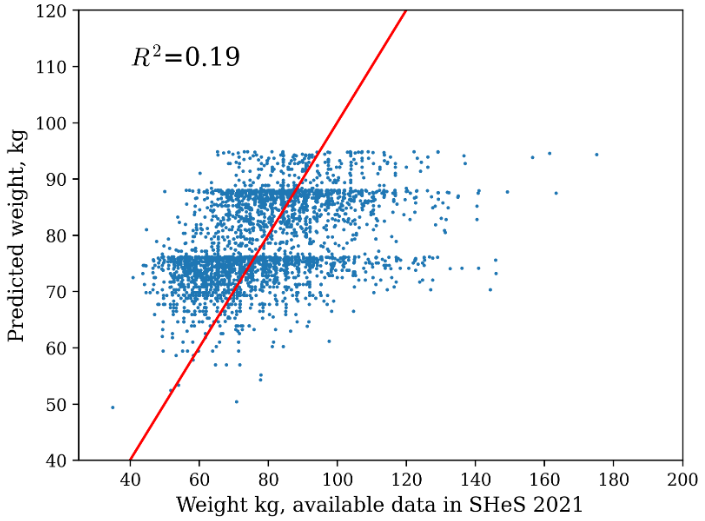
R^2=0.19
Predicted weight, kg
Weight kg, available data in SHeS 2021
120
110
100
90
80
70
60
50
40
40
60
80
100
120
140
160
180
200

(B)

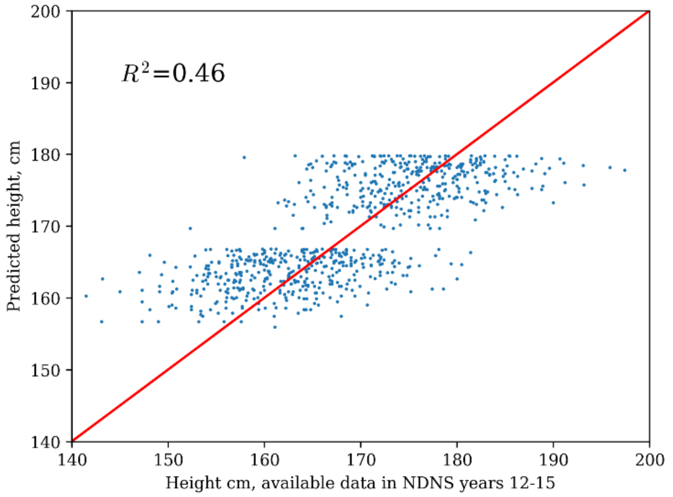
R^2=0.46
Predicted height, cm
Height cm, available data in NDNS years 12-15
200
190
180
170
160
150
140
140
150
160
170
180
190
200

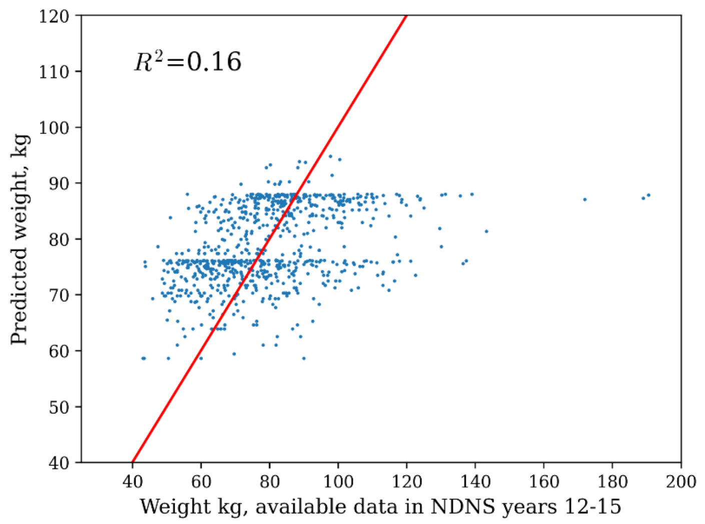

Figure S 18. Predicted height and weight compared to the height and weight data available in SHeS 2021 (n=3,151) (A) which was used as the training set for the BSR algorithm along with (B) the performance of the resultant imputation models on the out-of-sample dataset NDNS rounds 12-15 (n=772).

At the end of each simulation year each individual's age, systolic blood pressure, total cholesterol and HDL cholesterol were updated. Disease risk in each subsequent year was calculated according to these updated values as well as each individual's new BMI from the BMI model. Prevented cases of each health outcome were calculated by taking the difference between the yearly incidence in the baseline pathway with no meat or dairy reduction to that in each pathway with a meat and dairy reduction, before summing this difference over the ten simulation years. This process was then repeated for 50 iterations, resampling over sources of uncertainty. Sources of uncertainty include:

- the model parameters in the imputation models for systolic blood pressure, HDL cholesterol and total cholesterol,
- the relative risk associations between meat and dairy intake and disease risk and
- the relative risk of mortality for individuals with diabetes, CVD or both.
- MET scores associated with each physical activity variable

The choice of 50 iterations was motivated by the finding that the width of the uncertainty interval in the prevented cases of each disease did not substantially decrease beyond ~40 iterations, as shown in the case of prevented diabetes cases in the CCC 2030 pathway in **Figure S19**. To obtain uncertainty estimates for the health outcomes, both the lower 2.5% and the upper 97.5% were taken from the distribution of prevented cases over all simulation runs.

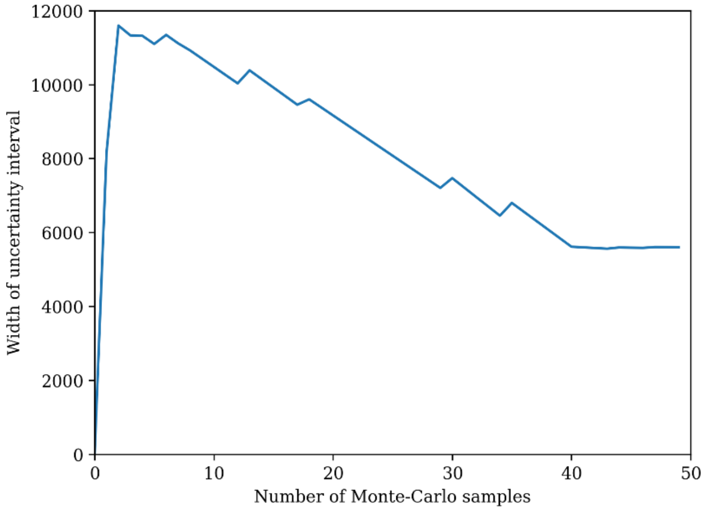

Figure S 19. Relationship between the width of the uncertainty interval in the prevented cases of diabetes over ten years in the CCC 2030 pathway with the number of Monte-Carlo iterations of the simulation. The width flattens at approximately 40 samples.

While the health outcome results in the main text account for the additional health impact associated with the combined effect of the reduction in BMI, in **Table S11** the health outcome results for prevented diabetes cases, CVD cases and all-cause mortalities over 10 years assuming isocaloric substitution by omitting the decrease in BMI from the yearly disease risk estimates. These results assume that the composition of foods that replace the reduced meat and dairy have no net effect on disease risk. These results can be compared to the no

replacement scenarios in the main text to provide estimates for the range of health outcomes to be expected in each of the reduction scenarios under different substitution scenarios across the Scottish adult population. In addition, by taking the ratio of the prevented cases in scenarios with isocaloric replacement compared to the prevented cases in the equivalent scenario with no replacement it is possible to obtain estimates for the relative impact on disease prevention from the daily reduction in calories versus the inherent change in risk from the reduced meat and dairy intake. In **Table S12** we provide the percentage of prevented cases of each health outcome in each pathway with no replacement that are attributable to weight loss. Uncertainty intervals were obtained by calculating the fraction of cases attributed to weight loss in each iteration of the simulation (calculated as one minus the ratio of the prevented cases in the isocaloric pathway to the equivalent no replacement pathway) and taking the bottom 2.5% and top 97.5% of the resultant distribution. Only 13.4% of the prevented diabetes cases over 10 years in the CCC 2030 scenario were a consequence of the change in risk associated with reducing meat and dairy, with the remainder of the health benefits associated with the long-term weight loss associated with the decrease in daily caloric intake. In the scenario with a 20% reduction in dairy alone, all the prevented diabetes cases were a consequence of weight loss with the isocaloric scenario showing an increase in diabetes prevalence relative to baseline due to the relative risk association between total dairy intake and type 2 diabetes risk being protective.

Table S11. Mean (95% uncertainty interval) impact of reducing meat and dairy from baseline (2021) levels assuming isocaloric replacement on chronic disease in a representative sample of adults 16+ years living in Scotland (n=3,447)..

**Pathway***	Climate Change Committee 2030	Climate Change Committee 2050	Scottish Dietary Goal (red meat 70g/day max)	Red meat 60g/day max	Red meat 31g/day max	Dairy reduction, 20%
**Health outcome**						
Prevented type-2 diabetes cases	5015 (2165, 7833)	10294 (5506, 14692)	8703 (5026, 12246)	11039 (6351, 15373)	20680 (10752, 29538)	-1716 (-3162, -657)
Prevented CVD cases	2427 (379, 4314)	4847 (1182, 8212)	2991 (733, 5161)	3938 (815, 6764)	8615 (1783, 15081)	-58 (-104, -22)
Prevented all-cause mortalities	301 (72, 511)	572 (177, 939)	324 (79, 562)	523 (151, 819)	1149 (665, 1597)	-34 (-63, -13)

*Pathways: 'Climate Change Committee 2030', a 20% reduction in all meat and dairy; 'Climate Change Committee 2050', a 35% reduction in all meat and 20% reduction in all dairy; 'Scottish Dietary Goal (red meat 70g/day max)', reducing red meat to a maximum intake of 70g/day and 20% reduction in all dairy; 'Red meat 60g/day max', reducing red meat to a maximum intake of 60g/day and 20% reduction in all dairy; and 'Red meat 31g/day max', reducing red meat to a maximum intake of 31g/day and 20% reduction in all dairy.

Table S12. Percentage (95% uncertainty interval) of prevented cases for each health outcome attributable to weight loss in the no-replacement scenarios following reduced meat and dairy intake from baseline (2021) levels in a representative sample of adults 16+ years living in Scotland (n=3,447).

**Pathway***	Climate Change Committee 2030	Climate Change Committee 2050	Scottish Dietary Goal (red meat 70g/day max)	Red meat 60g/day max	Red meat 31g/day max	Dairy reduction, 20%
**Health outcome**						
% prevented type-2 diabetes cases attributable to weight loss	86.6 (81.4, 93.0)	79.4 (74.2, 86.6)	78.0 (72.8, 84.6)	74.7 (69.4, 82.2)	65.6 (58.2, 76.6)	100**
% prevented CVD cases attributable to weight loss	76.0 (64.7, 94.7)	66.9 (54.9, 87.9)	71.8 (60.0, 90.1)	67.7 (55.3, 89.9)	56.0 (41.4, 84.2)	100**
% prevented all-cause mortalities attributable to weight loss	76.4 (66.0, 92.1)	68.1 (57.1, 85.8)	74.5 (62.9, 91.2)	65.0 (54.6, 85.1)	49.3 (41.2, 61.9)	100**

*Pathways: 'Climate Change Committee 2030', a 20% reduction in all meat and dairy; 'Climate Change Committee 2050', a 35% reduction in all meat and 20% reduction in all dairy; 'Scottish Dietary Goal (red meat 70g/day max)', reducing red meat to a maximum intake of 70g/day and 20% reduction in all dairy; 'Red meat 60g/day max', reducing red meat to a maximum intake of 60g/day and 20% reduction in all dairy; and 'Red meat 31g/day max', reducing red meat to a maximum intake of 31g/day and 20% reduction in all dairy.
** All prevented cases are attributable to weight loss.

References

1. The Scottish Health Survey 2021 - volume 2: technical report. https://www.gov.scot/publications/scottish-health-survey-2021-volume-2-technical-report/.
2. Amoutzopoulos, B. *et al.* Rationalisation of the UK Nutrient Databank for Incorporation in a Web-Based Dietary Recall for Implementation in the UK National Diet and Nutrition Survey Rolling Programme. *Nutrients* **14**, 4551 (2022).
3. Fitt, E. *et al.* Disaggregating composite food codes in the UK National Diet and Nutrition Survey food composition databank. *Eur J Clin Nutr* **64**, S32-36 (2010).
4. Jaacks, L. M. *et al.* Disaggregation of Dairy in Composite Foods in the United Kingdom. *Current Developments in Nutrition* **8**, 103774 (2024).
5. Lafrenière, J., Lamarche, B., Laramée, C., Robitaille, J. & Lemieux, S. Validation of a newly automated web-based 24-hour dietary recall using fully controlled feeding studies. *BMC Nutr* **3**, 34 (2017).
6. Page, P., Steer, T., Amoutzopoulos, B., Harvey, A. & Holmes, L. *Rebuild of the Food Standards Agency Recipes Database, Final Report*. http://doc.ukdataservice.ac.uk/doc/8159/mrdoc/pdf/8159_fsa_ recipes_database_rebuild_final_report.pdf (2015).
7. *The Seventh Carbon Budget, Advice for the UK Government*. https://www.theccc.org.uk/publication/the-seventh-carbon-budget/.
8. Harrington, R. A., Adhikari, V., Rayner, M. & Scarborough, P. Nutrient composition databases in the age of big data: foodDB, a comprehensive, real-time database infrastructure. *BMJ Open* **9**, e026652 (2019).
9. Clark, M. *et al.* Estimating the environmental impacts of 57,000 food products. *Proceedings of the National Academy of Sciences* **119**, e2120584119 (2022).
10. Poore, J. & Nemecek, T. Reducing food's environmental impacts through producers and consumers. *Science* **360**, 987–992 (2018).

11. McCance and Widdowson's The Composition of Foods Integrated Dataset 2021. https://www.gov.uk/government/publications/composition-of-foods-integrated-dataset-cofid (2021).
12. Lennox, A. *et al. Misreporting in the National Diet and Nutrition Survey Rolling Programme (NDNS RP): Summary of Results and Their Interpretation*. (2014).
13. *Government Dietary Recommendations*. https://assets.publishing.service.gov.uk/media/5a749fece5274a44083b82d8/government_dietary_recommendations.pdf.
14. National Records of Scotland. National Records of Scotland. *National Records of Scotland* https://www.nrscotland.gov.uk/statistics-and-data/statistics/statistics-by-theme/population/population-estimates/mid-year-population-estimates/mid-2021.
15. Lumley, T. *Complex Surveys: A Guide to Analysis Using R: A Guide to Analysis Using R*. (John Wiley and Sons, 2010).
16. Freedman, L. S., Guenther, P. M., Dodd, K. W., Krebs-Smith, S. M. & Midthune, D. The Population Distribution of Ratios of Usual Intakes of Dietary Components That Are Consumed Every Day Can Be Estimated from Repeated 24-Hour Recalls, ,. *The Journal of Nutrition* **140**, 111–116 (2010).
17. Krebs-Smith, S. M., Kott, P. S. & Guenther, P. M. Mean proportion and population proportion: Two answers to the same question? *Journal of the American Dietetic Association* **89**, 671–676 (1989).
18. Kennedy, J., Alexander, P., Taillie, L. S. & Jaacks, L. M. Estimated effects of reductions in processed meat consumption and unprocessed red meat consumption on occurrences of type 2 diabetes, cardiovascular disease, colorectal cancer, and mortality in the USA: a microsimulation study. *The Lancet Planetary Health* **8**, e441–e451 (2024).
19. Alva, M. L., Hoerger, T. J., Zhang, P. & Gregg, E. W. Identifying risk for type 2 diabetes in different age cohorts: does one size fit all? *BMJ Open Diabetes Res Care* **5**, e000447 (2017).
20. D'Agostino, R. B. *et al.* General Cardiovascular Risk Profile for Use in Primary Care. *Circulation* **117**, 743–753 (2008).

21. Yang, X. *et al.* Meat and fish intake and type 2 diabetes: Dose–response meta-analysis of prospective cohort studies. *Diabetes & Metabolism* **46**, 345–352 (2020).
22. Zhong, V. W. *et al.* Associations of Processed Meat, Unprocessed Red Meat, Poultry, or Fish Intake With Incident Cardiovascular Disease and All-Cause Mortality. *JAMA Internal Medicine* **180**, 503–512 (2020).
23. Feng, Y. *et al.* Consumption of Dairy Products and the Risk of Overweight or Obesity, Hypertension, and Type 2 Diabetes Mellitus: A Dose–Response Meta-Analysis and Systematic Review of Cohort Studies. *Advances in Nutrition* **13**, 2165–2179 (2022).
24. Team, N. R. of S. W. National Records of Scotland. *National Records of Scotland* https://www.nrscotland.gov.uk/statistics-and-data/statistics/statistics-by-theme/vital-events/general-publications/monthly-mortality-analysis-scotland (2013).
25. *Scottish Diabetes Survey*. https://www.diabetesinscotland.org.uk/wp-content/uploads/2023/02/Diabetes-Scottish-Diabetes-Survey-2021-final-version.pdf (2021).
26. Scottish heart disease statistics - Year ending 31 March 2022 - Scottish heart disease statistics - Publications - Public Health Scotland. https://publichealthscotland.scot/publications/scottish-heart-disease-statistics/scottish-heart-disease-statistics-year-ending-31-march-2022/.
27. Hall, K. D. *et al.* Quantification of the effect of energy imbalance on bodyweight. *The Lancet* **378**, 826–837 (2011).
28. Meh, K. *et al.* The dilemma of physical activity questionnaires: Fitter people are less prone to over reporting. *PLOS ONE* **18**, e0285357 (2023).
29. Welk, G. J., Beyler, N. K., Kim, Y. & Matthews, C. E. Calibration of Self-Report Measures of Physical Activity and Sedentary Behavior. *Medicine & Science in Sports & Exercise* **49**, 1473 (2017).
30. Sjostrom, M. *et al.* Guidelines for data processing analysis of the International Physical Activity Questionnaire (IPAQ) - Short and long forms. in (2005).
31. Kim, Y., Park, I. & Kang, M. Convergent validity of the International Physical Activity Questionnaire (IPAQ): meta-analysis. *Public Health Nutr* **16**, 440–452 (2013).

32. Doherty, A. *et al.* Large Scale Population Assessment of Physical Activity Using Wrist Worn Accelerometers: The UK Biobank Study. *PLOS ONE* **12**, e0169649 (2017).

33. Zhou, W. *et al.* Comparison of Pre-Diagnosis Physical Activity and Its Correlates between Lung and Other Cancer Patients: Accelerometer Data from the UK Biobank Prospective Cohort. *Int J Environ Res Public Health* **20**, 1001 (2023).

34. Ainsworth, B. E. *et al.* 2011 Compendium of Physical Activities: A Second Update of Codes and MET Values. *Medicine & Science in Sports & Exercise* **43**, 1575 (2011).

35. Kozey, S., Lyden, K., Staudenmayer, J. & Freedson, P. Errors in MET Estimates of Physical Activities Using 3.5 ml·kg−1·min−1 as the Baseline Oxygen Consumption. *Journal of Physical Activity and Health* **7**, 508–516 (2010).

36. Gerrior, S., Juan, W. & Basiotis, P. An Easy Approach to Calculating Estimated Energy Requirements. *Prev Chronic Dis* **3**, (2006).

37. Mifflin, M. *et al.* A new predictive equation for resting energy expenditure in healthy individuals. *The American Journal of Clinical Nutrition* **51**, 241–247 (1990).

38. Guimerà, R. *et al.* A Bayesian machine scientist to aid in the solution of challenging scientific problems. *Science Advances* **6**, eaav6971 (2020).

39. Negri, V., Vázquez, D., Sales-Pardo, M., Guimerà, R. & Guillén-Gosálbez, G. Bayesian Symbolic Learning to Build Analytical Correlations from Rigorous Process Simulations: Application to CO2 Capture Technologies. *ACS Omega* **7**, 41147–41164 (2022).

40. Vázquez, D., Guimerà, R., Sales-Pardo, M. & Guillén-Gosálbez, G. Automatic modeling of socioeconomic drivers of energy consumption and pollution using Bayesian symbolic regression. *Sustainable Production and Consumption* **30**, 596–607 (2022).

41. Hoffman, M. D. & Gelman, A. The No-U-Turn Sampler: Adaptively Setting Path Lengths in Hamiltonian Monte Carlo. *Journal of Machine Learning Research* **15**, (2014).

42. Abril-Pla, O. *et al.* PyMC: a modern, and comprehensive probabilistic programming framework in Python. *PeerJ Comput. Sci.* **9**, e1516 (2023).
