# Supplementary material for: Reduced meat and dairy consumption improves health, environmental and most nutritional outcomes without increasing diet costs among Scottish adults
Source: Nat Food. 2026 Jul 3;7(7):711–21. doi: 10.1038/s43016-026-01384-3 (PMC13388101; doi:10.1038/s43016-026-01384-3)
Supplement: Supplementary file 2 — Reporting Summary [file 43016_2026_1384_MOESM2_ESM.pdf]

nature portfolio

Corresponding author(s): Joe Kennedy

Last updated by author(s): Mar 25, 2026

Reporting Summary

Nature Portfolio wishes to improve the reproducibility of the work that we publish. This form provides structure for consistency and transparency in reporting. For further information on Nature Portfolio policies, see our Editorial Policies and the Editorial Policy Checklist.

Statistics

For all statistical analyses, confirm that the following items are present in the figure legend, table legend, main text, or Methods section.

n/a	Confirmed	
☐	☒	The exact sample size (*n*) for each experimental group/condition, given as a discrete number and unit of measurement
☒	☐	A statement on whether measurements were taken from distinct samples or whether the same sample was measured repeatedly
☒	☐	The statistical test(s) used AND whether they are one- or two-sided<br>*Only common tests should be described solely by name; describe more complex techniques in the Methods section.*
☐	☒	A description of all covariates tested
☒	☐	A description of any assumptions or corrections, such as tests of normality and adjustment for multiple comparisons
☐	☒	A full description of the statistical parameters including central tendency (e.g. means) or other basic estimates (e.g. regression coefficient) AND variation (e.g. standard deviation) or associated estimates of uncertainty (e.g. confidence intervals)
☒	☐	For null hypothesis testing, the test statistic (e.g. *F*, *t*, *r*) with confidence intervals, effect sizes, degrees of freedom and *P* value noted<br>*Give P values as exact values whenever suitable.*
☐	☒	For Bayesian analysis, information on the choice of priors and Markov chain Monte Carlo settings
☒	☐	For hierarchical and complex designs, identification of the appropriate level for tests and full reporting of outcomes
☒	☐	Estimates of effect sizes (e.g. Cohen's *d*, Pearson's *r*), indicating how they were calculated

Our web collection on statistics for biologists contains articles on many of the points above.

Software and code

Policy information about availability of computer code

Data collection	No software was used for data collection
Data analysis	All code that was used in the analysis is publicly available at DOI: 10.5281/zenodo.20447123

For manuscripts utilizing custom algorithms or software that are central to the research but not yet described in published literature, software must be made available to editors and reviewers. We strongly encourage code deposition in a community repository (e.g. GitHub). See the Nature Portfolio guidelines for submitting code & software for further information.

Data

Policy information about availability of data

All manuscripts must include a data availability statement. This statement should provide the following information, where applicable:

- Accession codes, unique identifiers, or web links for publicly available datasets
- A description of any restrictions on data availability
- For clinical datasets or third party data, please ensure that the statement adheres to our policy

The Scottish Health Survey 2021 data can be obtained from the UK Data Service (https://ukdataservice.ac.uk/, doi: 10.5255/UKDA-SN-9048-2). Due to legal constraints, product level data in foodDB and the mapping between the nutrient databank and foodDB are not publicly available. Access for the purpose of replication can be requested from trisha.gordon@ndph.ox.ac.uk. Data on the estimated environmental impacts and costs for food items in the UK Nutrient Databank derived from foodDB is available via Edinburgh DataShare (Reference 50).

Research involving human participants, their data, or biological material

Policy information about studies with human participants or human data. See also policy information about sex, gender (identity/presentation), and sexual orientation and race, ethnicity and racism.

Reporting on sex and gender	Results were broken down by the "Sex" variable in the Scottish Health Survey which records self-reported sex by the household respondent. More details on data collection in the Scottish Health Survey 2021 can be found at https://datacatalogue.ukdataservice.ac.uk/studies/study/9048#documentation
Reporting on race, ethnicity, or other socially relevant groupings	Results were broken down by Scottish Index of Multiple Deprivation quintiles to assess any differences by deprivation quintile. These data were assigned via the postcode of the respondent households. Full details on data collection in the 2021 Scottish Health Survey can be found at https://datacatalogue.ukdataservice.ac.uk/studies/study/9048#documentation
Population characteristics	See above
Recruitment	See documentation on the 2021 Scottish Health Survey: https://datacatalogue.ukdataservice.ac.uk/studies/study/9048#documentation
Ethics oversight	No ethical approval was needed for this study as all primary data on participants are publicly available.

Note that full information on the approval of the study protocol must also be provided in the manuscript.

Field-specific reporting

Please select the one below that is the best fit for your research. If you are not sure, read the appropriate sections before making your selection.

☐ Life sciences ☒ Behavioural & social sciences ☐ Ecological, evolutionary & environmental sciences

For a reference copy of the document with all sections, see nature.com/documents/nr-reporting-summary-flat.pdf

Behavioural & social sciences study design

All studies must disclose on these points even when the disclosure is negative.

Study description	Quantitative study
Research sample	Nationally representative survey data of adults (16+) living in Scotland in 2021.
Sampling strategy	The Scottish Health Survey ensure that the sampling is nationally representative (https://datacatalogue.ukdataservice.ac.uk/studies/study/9048#details)
Data collection	More information on the data collection methods can be found in the SHeS 2021 documentation: https://datacatalogue.ukdataservice.ac.uk/studies/study/9048#documentation
Timing	Time period of data collection can be obtained in the SHeS 2021 documentation: https://datacatalogue.ukdataservice.ac.uk/studies/study/9048#documentation
Data exclusions	Pregnant participants (N=16) were excluded from the health simulations as the disease risk models used in the study are not applicable to pregnant women.
Non-participation	Full details on how the Scottish Health Survey 2021 data were collected can be found at https://datacatalogue.ukdataservice.ac.uk/studies/study/9048#documentation
Randomization	Full details on how the Scottish Health Survey 2021 data were collected can be found at https://datacatalogue.ukdataservice.ac.uk/studies/study/9048#documentation

Reporting for specific materials, systems and methods

We require information from authors about some types of materials, experimental systems and methods used in many studies. Here, indicate whether each material, system or method listed is relevant to your study. If you are not sure if a list item applies to your research, read the appropriate section before selecting a response.

Materials & experimental systems

n/a	Involved in the study
☒	☐ Antibodies
☒	☐ Eukaryotic cell lines
☒	☐ Palaeontology and archaeology
☒	☐ Animals and other organisms
☒	☐ Clinical data
☒	☐ Dual use research of concern
☒	☐ Plants

Methods

n/a	Involved in the study
☒	☐ ChIP-seq
☒	☐ Flow cytometry
☒	☐ MRI-based neuroimaging

Plants

Seed stocks

Report on the source of all seed stocks or other plant material used. If applicable, state the seed stock centre and catalogue number. If plant specimens were collected from the field, describe the collection location, date and sampling procedures.

Novel plant genotypes

Describe the methods by which all novel plant genotypes were produced. This includes those generated by transgenic approaches, gene editing, chemical/radiation-based mutagenesis and hybridization. For transgenic lines, describe the transformation method, the number of independent lines analyzed and the generation upon which experiments were performed. For gene-edited lines, describe the editor used, the endogenous sequence targeted for editing, the targeting guide RNA sequence (if applicable) and how the editor was applied.

Authentication

Describe any authentication procedures for each seed stock used or novel genotype generated. Describe any experiments used to assess the effect of a mutation and, where applicable, how potential secondary effects (e.g. second site T-DNA insertions, mosiacism, off-target gene editing) were examined.
